# Low-oxygen waters limited habitable space for early animals

**DOI:** 10.1038/ncomms12818

**Published:** 2016-09-23

**Authors:** R. Tostevin, R. A. Wood, G. A. Shields, S. W. Poulton, R. Guilbaud, F. Bowyer, A. M. Penny, T. He, A. Curtis, K. H. Hoffmann, M. O. Clarkson

**Affiliations:** 1 Department of Earth Sciences, University College London, Gower Street, London WC1E 6BT, UK; 2 School of GeoSciences, The University of Edinburgh, James Hutton Road, Edinburgh EH9 3FE, UK; 3 School of Earth and Environment, University of Leeds, Leeds LS2 9JT, UK; 4 Department of Earth Sciences, University of Cambridge, Downing Street, Cambridge CB2 3EQ, UK; 5 Geological Survey of Namibia, Private Bag 13297, Windhoek, Namibia; 6 Department of Chemistry, University of Otago, Dunedin 9054, New Zealand

## Abstract

The oceans at the start of the Neoproterozoic Era (1,000–541 million years ago, Ma) were dominantly anoxic, but may have become progressively oxygenated, coincident with the rise of animal life. However, the control that oxygen exerted on the development of early animal ecosystems remains unclear, as previous research has focussed on the identification of fully anoxic or oxic conditions, rather than intermediate redox levels. Here we report anomalous cerium enrichments preserved in carbonate rocks across bathymetric basin transects from nine localities of the Nama Group, Namibia (∼550–541 Ma). In combination with Fe-based redox proxies, these data suggest that low-oxygen conditions occurred in a narrow zone between well-oxygenated surface waters and fully anoxic deep waters. Although abundant in well-oxygenated environments, early skeletal animals did not occupy oxygen impoverished regions of the shelf, demonstrating that oxygen availability (probably >10 μM) was a key requirement for the development of early animal-based ecosystems.

## Abstract (web summary)

The importance of oxygen in supporting early animal ecosystems is unclear because most proxies are unable to distinguish well-oxygenated from intermediate waters. Here, the authors show that early skeletal animals were restricted to well-oxygenated habitats, suggesting they had a high metabolic oxygen demand.

## Graphical abstract

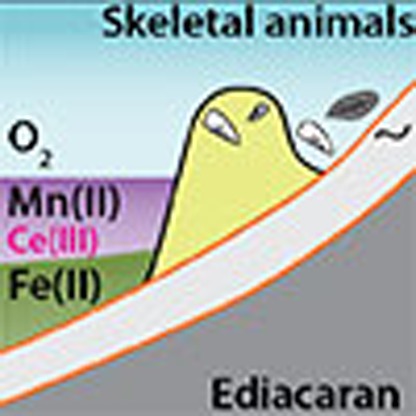

Geochemical proxies based on Fe-S-C and trace metal systematics have been widely used to reconstruct the progressive oxygenation of the oceans during the Neoproterozoic and Cambrian^1^,^2^,^3^,^4^,^5^,^6^,^7^. Accumulating evidence indicates that the deep oceans were dominantly anoxic and ferruginous (Fe containing) throughout most of the Precambrian, with euxinic (sulfidic) mid-depth waters prevalent along continental margins from ∼1.8 to 1.0 billion years ago (Ga)^1^,^6^,^8^. From ∼1.0 to 0.58 Ga, however, euxinic mid-depth waters became less prevalent and ferruginous conditions expanded, with oxic conditions still largely restricted to surface waters^1^,^8^,^9^. The oxygenation of the deeper marine realm was both protracted and spatially heterogeneous, with some marine basins recording persistent deep-water oxygenation from ∼580 Ma, whereas regional anoxia remained a feature of some deeper shelf environments into the Cambrian, ∼520 Ma (refs 4, 5, 7, 10) and beyond.

The course of Neoproterozoic oxygenation, and cause and effect associations with the appearance of animals, remains controversial^4^,^11^,^12^,^13^. Although modern soft-bodied sponge-grade animals may tolerate oxygen concentrations as low as 1.25–10 μM^14^, new innovations in the late Ediacaran, such as motility^15^, the rise of predation and skeletonization^16^,^17^,^18^, are all hypothesized to have required higher levels of oxygen^19^. However, the oxygen demands of early animals are unconstrained and observations from modern biota cannot necessarily be applied to early animals of unknown affinity. Furthermore, although soft-bodied and skeletal Ediacaran fauna dominantly occur in sediments interpreted to have been deposited from oxic waters, fossil occurrences have also been reported in sediments characterized by anoxic geochemical signals^5^,^20^. In the latter case, this may be because some early complex organisms were able to colonize habitats during fleeting periods of oxia (such short-lived oxygenation is difficult to detect by geochemical proxies that tend to integrate relatively long periods of time). In both of the above cases, however, there is uncertainty as to whether early animal evolution occurred under fully oxygenated conditions or whether intermediate redox conditions were more prevalent, which by extension suggests that the oxygen requirements of more complex organisms were lower^3^,^14^. An in-depth understanding of these links is currently hampered by the inability of most redox proxies to distinguish between fully oxygenated and intermediate redox states, including nitrogenous or manganous conditions, which may overlap with low concentrations of oxygen^21^,^22^. Indeed, it is possible that ‘oxic' horizons identified through Fe and trace element geochemistry may in fact have formed under low-oxygen conditions (but not fully anoxic), at levels insufficient to support diverse skeletal animal communities.

In oxic environments, Ce(III) is oxidized to insoluble Ce(IV) and preferentially scavenged relative to the rest of the rare earth elements and yttrium, REY^23^. The standard reduction potential of Ce(IV) (+1.61°V) is closer to Mn(IV) (+1.23°V) than Fe(III) (+0.77°V) and Ce oxidation is catalysed on the surface of Mn (oxyhydr)oxides^24^. Therefore, the redox cycling of Ce in seawater is closely related to Mn(II)/Mn(IV) transformations, which occur at a higher redox potential than the Fe(II)/Fe(III) couple, and hence Mn cycling is more sensitive to intermediate redox conditions^23^,^24^,^25^,^26^. Ce anomalies (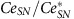) are calculated here based on relative enrichments or depletions in shale-normalized Ce ([Ce]_SN_) compared with neighbouring non-redox sensitive REY:


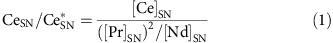


Owing to the accumulation of Ce(IV) on the surface of Mn (oxyhydr)oxides, oxic seawater becomes Ce depleted and exhibits a negative Ce anomaly (<0.9)^23^. These Mn (oxyhydr)oxides may be buried intact in sediments beneath oxic bottom waters, or may dissolve in the water column if they encounter low-oxygen waters, releasing excess Ce. Therefore, waters beneath the Mn(IV)/Mn(II) redoxcline commonly exhibit a positive Ce anomaly (>1.3)^25^,^26^,^27^. Positive Ce anomalies have been recorded alongside Mn enrichments in some modern waters, including Lake Vanda, Antarctica (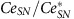 up to 2.3, Fig. 1)^25^, in anoxic brines in the eastern Mediterranean (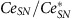 up to 2.43, Fig. 1)^27^ and in the deep-marine Cariaco Basin (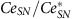 up to 1.21)^26^.

Water column REY and associated Ce anomalies are thought to be preserved in non-skeletal carbonate rocks without fractionation^28^. Carbonate-bound REY are relatively robust to diagenetic alteration^28^ and dolomitization^29^, but any alteration of the Ce anomaly can be identified using non-redox sensitive REY anomalies, such as the Y/Ho ratio, which would also be altered away from seawater patterns^30^,^31^. Sequential dissolution methods enable REY in the carbonate phase to be isolated, preventing contributions from sedimentary (oxyhydr)oxides or clays^31^, which would carry a non-seawater signature.

In the present study, we measured REY in 259 carbonate rocks of the Nama Group from nine sites across two basins. The majority of samples are very pure calcites with low siliciclastic components, but where samples are partially dolomitized they have been treated differently during leaching^31^. The resulting REY data have been screened for traditional seawater features (Y/Ho ratios >67) and samples with evidence for diagenetic alteration or contributions from non-carbonate phases have been excluded from the presented Ce/Ce* data (see Methods). We additionally use redox interpretations based on published Fe speciation data for these carbonate samples^5^. Fe speciation distinguishes anoxic from oxic water column conditions through enrichments in highly reactive Fe (Fe_HR_) relative to total Fe (Fe_T_)^1^,^32^. Anoxic enrichments in Fe_HR_ occur due to the water column formation of either pyrite under euxinic conditions^32^ or non-sulfidized Fe_HR_ minerals (such as Fe oxides or carbonates) under anoxic ferruginous conditions^1^ (see Methods). We interpret ususual Ce enrichments across the Nama Group to indicate Mn-rich, low-oxygen conditions, supported by additional redox information from Fe-based proxies on the same samples. This enables us to distinguish fully anoxic, intermediate and well-oxygenated waters across a shelf-to-basin transect and compare these with the distribution of early skeletal animal life.

## Results

### Geological setting

The succession was deposited ∼550–541 Ma broadly coincident with the first appearance of skeletal animals^16^,^17^,^18^, as well as trace fossil evidence for motility^15^ and soft-bodied fossils belonging to the Ediacaran biota^33^. Our samples cover a range of palaeo depths from shallow inner ramp to deeper outer ramp waters, in the Kanies, Omkyk and Hoogland Members of the Kuibis Subgroup, and the Spitzkopf and Feldschuhorn Members of the upper Schwarzrand Subgroup^5^,^16^ (Fig. 2, Supplementary Table 1 and also see Supplementary Note 1 for full details of the geological setting). We focus on the first known skeletal animals, *Cloudina*, a globally distributed eumetazoan of possible cnidarian affinity^17^,^34^,^35^; *Namacalathus*, interpreted as a stem group eumetaozan^36^ or triploblast lophophorate^37^ and reported from multiple localities; and *Namapoikia*, an encrusting possible cnidarian or poriferan known only from the Nama Group^18^.

### Ce anomaly interpretation

In the Nama Group, the majority of REY distribution patterns are smooth and show a flat or light REY-depleted shape on shale-normalized plots, positive La anomalies, low total rare earth elements (REE) concentrations and superchondritic Y/Ho ratios (>67), all of which indicate preservation and extraction of original seawater signals (see Supplementary Note 2 and supplementary Figs 1–10 for a description of all data). Four samples exhibit negative Ce anomalies (<0.9; Fig. 2), consistent with an oxic water column interpretation obtained for these samples from Fe speciation^5^. Ce anomalies are, as expected, absent from the persistently anoxic and ferruginous deepest water setting^5^ (Fig. 2). However, significant positive Ce anomalies (1.30–2.15) are prevalent in inner ramp sections in both sub-basins (64 samples). In six cases, positive Ce anomalies are associated with anoxic ferruginous signals and in one case a positive Ce anomaly is associated with a sample that gives a robust oxic Fe_HR_/Fe_T_ signal. However, for the majority of samples (∼90%), Fe_T_ was <0.5 wt%, preventing a robust evaluation of water column redox conditions from Fe speciation alone. In these cases, samples have elevated Mn/Fe ratios (median=0.39), when compared with samples with no positive Ce anomalies (median=0.14) and anoxic ferruginous samples (median=0.10), which provides an independent constraint on water column redox conditions, as discussed below (Fig. 3).

**Figure 1:**
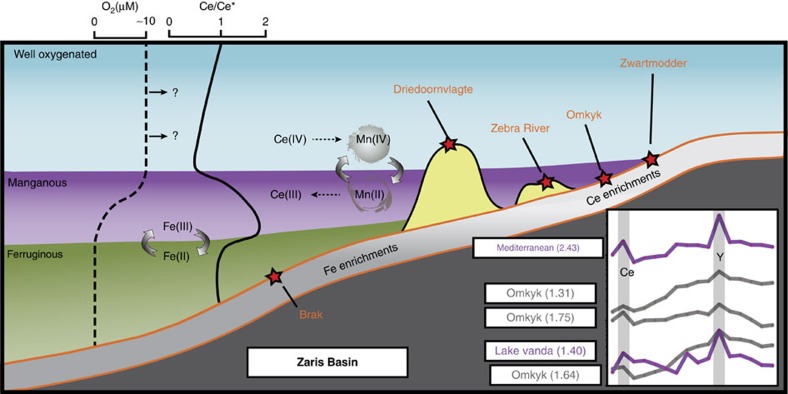
Schematic representation of redox zones and associated geochemical signals. Generalized redox conditions across the Zaris Basin, Nama Group, during a highstand systems tract. Positive Ce anomalies form as Mn (oxyhydr)oxides dissolve in the manganous zone and Fe enrichments form under anoxic ferruginous conditions. Ten micromole is an estimate of O_2_ concentrations in the manganous zone, but overlying well-oxygenated waters probably contained higher O_2_ concentrations. Representative REY patterns, including positive Ce anomalies (magnitude in brackets), are shown for the Omkyk section in the Nama Group, alongside manganous zones from two modern environments^25^,^27^ (modern water column data plotted as [REY] × 10^6^ for easy comparison with sedimentary [REY]).

The regionally widespread positive Ce anomalies across the Zaris and Witputs Basins of the Nama Group imply a surplus of Ce sustained by a rain down of Mn (oxyhydr)oxides from shallow oxygenated surface waters and this is supported by the elevated Mn/Fe ratios of these samples (Fig. 3). Redox cycling of Mn (oxyhydr)oxides across the Mn(IV/II) redoxcline would leave ambient waters locally enriched in the Ce released during Mn(IV) reduction (Fig. 1). We therefore interpret positive Ce anomalies (>1.3) to indicate intermediate manganous conditions (Table 1 and also see Supplementary Discussion for alternative Ce enrichment mechanisms). Where there is an absence of both positive Ce anomalies and any indication of enrichment in Fe (that is, Fe_HR_/Fe_T_<0.22 or Fe_T_<0.5 wt%), we suggest that bottom waters were probably well oxygenated (which is consistent with Fe_HR_/Fe_T_ signals in interbedded siliciclastics^5^), thus preventing the onset of both Fe and Mn reduction. Where data are equivocal (for example, Fe_HR_/Fe_T_ between 0.22–0.38 and no Ce anomaly), we are unable to interpret redox conditions.

Positive Ce anomalies have not been widely reported from carbonate-rich sediments, but there are limited examples from iron formation^38^ and cherts^39^ in the earlier Paleozoic. Positive Ce anomalies, between 1.3 and 2.2, are also reported for late Ediacaran dolomites, from just a few samples in the possibly contemporaneous Krol Formation of northern India^40^. If these data were demonstrated to preserve seawater REY patterns, this strengthens the data from the Nama Group and hints that manganous conditions may have been a common feature of Ediacaran oceanic margins. By contrast, demonstrably contemporaneous terminal Ediacaran Ce anomaly data from the Yangtze platform, South China, show increasing Ce depletion towards the Ediacaran–Cambrian Boundary, indicating progressive oxygenation of the local marine environment^41^. Ce anomalies record local redox conditions and thus independent signals would be expected both within and between marine basins.

### Redox conditions in the Nama group

The outer ramp was persistently anoxic and ferruginous (Brak section), and animals are absent from these settings^5^ (Fig. 4). The deep inner-ramp sections show periods of anoxic ferruginous, manganous and well-oxygenated conditions (Zebra River and Omkyk sections). In these settings, animals are notably absent from ferruginous and manganous waters, whereas well-oxygenated waters support abundant skeletal animals, up to 35 mm in diameter, and adjacent localities show trace fossil evidence for motility^15^. The shallowest inner ramp sections show high-frequency temporal fluctuations between anoxic ferruginous, manganous and well-oxygenated conditions (Zwartmodder, Arasab and Grens sections), as might be expected due to fluctuations in the depth of the chemocline (Fig. 4). At Zwartmodder, skeletal animals are present in thin beds^5^, but there is only one skeletal horizon at Grens and no animal fossils at Arasab.

In contrast to these ecologies, the Driedoornvlagte pinnacle reef grew within a transgressive systems tract in a mid-ramp position, which was persistently well-oxygenated and hosts some very large skeletal animals^5^,^18^ (up to 1 m) and complex reef-building ecologies^17^. In the younger Schwarzrand Subgroup, which extends close to the Ediacaran–Cambrian Boundary (∼547–541 Ma), there is evidence for persistent well-oxygenated conditions^5^ and mid-ramp Pinnacle Reefs host mixed communities of large and small skeletal animals. At Swartpunt, abundant burrows and soft-bodied biota occur in siliciclastic horizons, where Fe speciation indicates oxic conditions^5^, whereas small *in-situ* skeletal animals are found in carbonate rocks throughout the succession^5^.

## Discussion

Our geochemical and palaeontological data demonstrate a striking relationship between the precise redox condition of the water column and the presence and abundance of evidence for animal life. Constraints from the modern open ocean suggest that dissolved Mn(II), and therefore Ce(III), can start to build up in low concentrations in oxic waters with dissolved O_2_<100 μM^22^. However, manganous conditions, whereby Mn becomes the dominant redox buffer, are achieved at lower oxygen concentrations. Reduced Mn can remain stable in the presence of up to 10 μM O_2_ (refs 21, 42), although Mn oxidation has been reported locally at lower O_2_ concentrations where oxidation is catalysed by enzymatic processes^43^. Thus, active Mn cycling can occur in anoxic waters, but is commonly documented in partially oxic waters with at least 10 μM O_2_ (and up to 100 μM O_2_; Fig. 1)^21^,^42^,^44^,^45^, which represents significant oxygen depletion in comparison with modern fully oxygenated surface waters (∼250 μM O_2_). The reduction potential for Ce is higher than that for Mn and so the 10 μM O_2_ constraint for manganous waters may represent a lower limit on Ce cycling, as sufficient O_2_ to oxidize both Ce and Mn is required for the formation of Ce anomalies.

Our multi-proxy approach allows us to distinguish between fully anoxic and intermediate waters, which contained low but significant amounts of oxygen. Where Fe speciation in Ce-enriched samples gives a robust anoxic signal (Fe_HR_/Fe_T_>0.38), Mn reduction may have persisted, but conditions must have been fully anoxic. However, the majority of samples interpreted to be manganous have insufficient Fe_T_ for Fe speciation (with 85% of these falling below 0.25 wt% Fe_T_ and 35% falling below 0.1 wt% Fe_T_). Even very low oxygen concentrations (nM) are sufficient to prevent Fe_HR_ enrichments and thus the low Fe_T_ in shallower environments across the Nama Group may be indicative of oxic conditions^46^, and this is supported by persistent oxic Fe_HR_/Fe_T_ ratios obtained from interbedded siliciclastics in some sections^5^. We therefore suggest that the manganous zone occurred between well-oxygenated surface waters and deeper anoxic, ferruginous waters, commonly overlapping with low but significant concentrations of oxygen (at least ∼10 μM; Fig. 1).

Oxygen exerts an important control on ecosystem structure in modern environments, whereby low-oxygen environments are inhabited by smaller animals often lacking skeletons and forming low-diversity communities with simple food webs^19^. In general, skeletons are absent from modern oxygen minimum zones when O_2_ drops below 13 μM and large animals are often absent below 45 μM (refs 47, 48). However, the importance of oxygen in supporting early animal ecosystems as they became increasingly complex in form, metabolic demand and behaviour through the Ediacaran Period is currently unresolved^2^,^3^,^5^,^11^,^12^,^13^. In the Nama Group the majority of small skeletal animals (>75%) and all evidence for large skeletal animals, motility, soft-bodied biota and complex or long-lived ecologies^17^ are found in sediments deposited from well-oxygenated waters (Fig. 4). The identification of low-oxygen, manganous water column conditions thus provides a compelling explanation for the general absence of biota in these settings and implies that poorly oxygenated conditions were insufficient to meet the relatively high oxygen requirements of these early skeletal animals^5^,^15^,^17^,^33^. If we take an upper O_2_ limit for Mn and Ce reduction of 10 μM O_2_, this suggests that Mn-enriched waters could theoretically support small, soft-bodied animals such as sponges^14^. In contrast, the absence of skeletal animals in Mn-enriched waters is consistent with the high energetic cost of skeletonization. Possible biomarkers for sponge animals appear in the fossil record at >635 Ma (ref. 49), but it is possible that the availability of well-oxygenated habitats was necessary to support the later appearance of skeletonization, at ∼550 Ma. However, it is also unlikely to be that reaching an oxygenation threshold alone is sufficient to explain the appearance of skeletons^50^ and many have argued that the trigger for the rise of skeletonization may have been ecological, such as the rise of predation^17^,^36^,^51^.

Our approach highlights that intermediate redox conditions were probably widespread in the Ediacaran ocean, but have not previously been appreciated due to the inability of most commonly used proxies to identify such conditions. Our data suggest that low-oxygen water column conditions were insufficient to support early skeletal and reef-building animals, and thus the extent of suitable habitat space may have been less than previously identified. The widespread radiation of skeletal animals during the subsequent Cambrian explosion may have been facilitated by a global rise in the extent of habitable, oxygenated seafloor^7^, alongside other genetic and ecological factors. Our data therefore yield new insight into the debate on the role of oxygen in early animal evolution, suggesting that well-oxygenated waters were necessary to support the appearance of the skeletal animals and complex ecologies that are typical of the terminal Neoproterozoic.

## Methods

### Geological context and sample quality

The Nama Group is a well-preserved terminal Neoproterozoic carbonate and siliciclastic sequence, ranging from upper shore-line/tidal flats to below-wave-base lower shoreface, deposited in a ramp system ∼550–541 Ma (refs 5, 16, 52, 53, 54, 55). Samples from nine shelf-to-basin sections within the Zaris and Witputs basins of the Nama Group encompass a range of palaeo-depths from outer- to inner-ramp settings (Supplementary Table 1). Stratigraphic correlations are well-established based on sequence boundaries and ash beds^5^,^16^. The age of the upper Nama Group is relatively well-constrained from U-Pb dating of three ash beds within the group, including one at 548.8±1 Ma in the Hoogland Member of the Kuibis Subgroup^54^, revised to 547.32±0.31 Ma (ref. 56). The base of the Nama Group is diachronous, but is between 553 and 548 Ma. The Proterozoic–Cambrian boundary is represented by a regionally extensive erosional unconformity near the top of the Schwarzrand Subgroup in the southern Basin^53^,^54^,^57^,^58^, which is overlain by incised-valley fill dated (U-Pb on an ashbed) at 539±1 Ma (ref. 54). Therefore, the Nama Group section spans at least 7 Myr and extends to within 2 Myr of the Ediacaran–Cambrian Boundary^52^.

Unweathered samples were selected and powdered or drilled avoiding alteration, veins or weathered edges. For Zebra River section, powders were drilled from thin section counterparts, targeting fine-grained cements. Carbonate rocks in the Nama Group are very pure, but they have all undergone pervasive recrystallization. Less than 15% of the samples in this study are dolomitized and there is no petrographic evidence for deep burial dolomitization in the Nama Group^5^,^55^.

**Figure 2:**
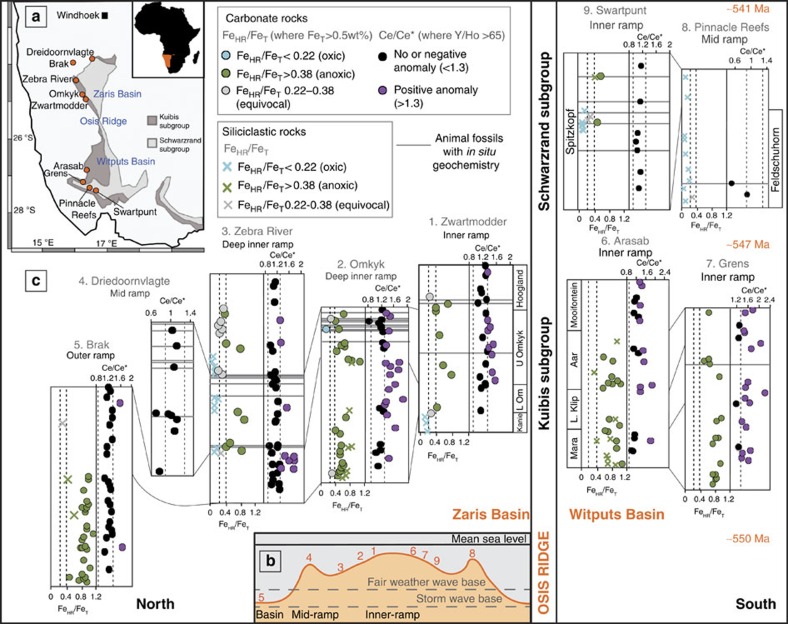
Summary of Ce_SN_/Ce*_SN_ and Fe-speciation data for nine localities. The location of nine sections within the Kuibis and Schwarzrand Subgroups of the Nama Group is shown on a simplified geological map of Namibia^52^,^53^,^55^,^57^ (**a**), as well as on a schematic cross-section, indicating average relative water depth (**b**). The numbers along the basin profile relate to the relative position of each section as numbered in **c**. Fe_HR_/Fe_T_ data for each location is shown for carbonate and siliciclastic rocks^5^, alongside 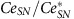 data, screened for carbonate rocks showing seawater REY patterns (for example, molar Y/Ho>67) (**c**). Blue Fe_HR_/Fe_T_ data indicate where Fe speciation predicts oxic conditions^5^ and positive Ce anomalies indicate where waters are interpreted to have been manganous. The presence of *in situ* biota is noted by grey lines^5^.

**Figure 3:**
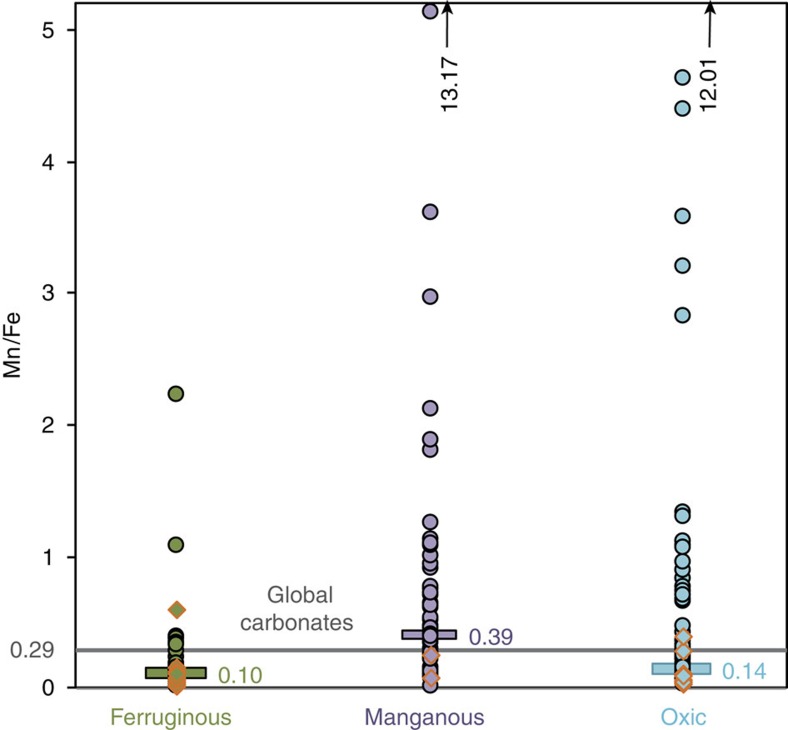
Average Mn/Fe ratios under different redox conditions. Mn/Fe ratios for samples identified as manganous (positive Ce/Ce* and low Fe_T_ or oxic Fe-speciation signals), ferruginous (anoxic Fe-speciation signals) and oxic (oxic Fe-speciation signals, no positive Ce anomaly). Mn/Fe is enriched in manganous samples compared with global carbonate (0.29; ref. 70). Bars represent median values. Red outlines indicate dolomitized samples. Arrows indicate samples with exceptionally high Mn/Fe ratios that lie above the limit of the *y* axis.

**Figure 4:**
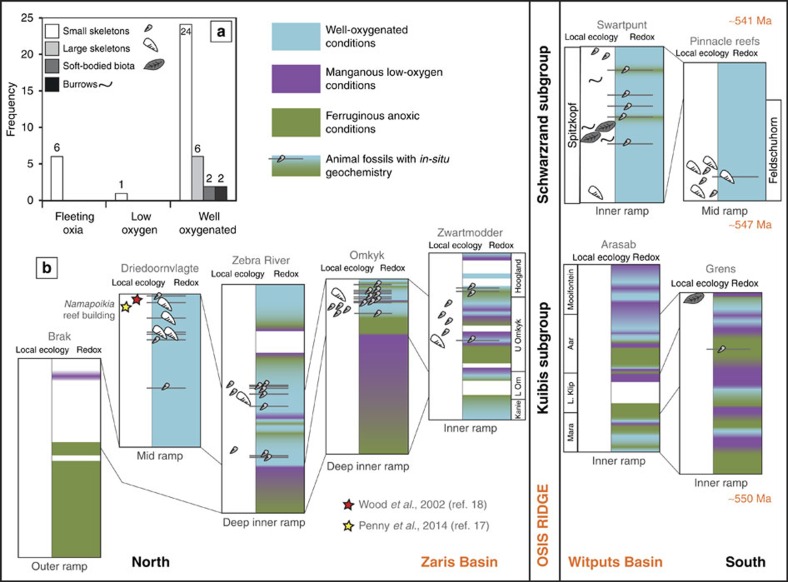
Integrated redox interpretations compared with local ecology. A comprehensive redox interpretation is shown for each of the nine localities in the Nama Group, determined using combined Fe and Ce signals. *In situ* fossils (grey lines), and local ecologies^5^ and general ecology from the literature^17^,^18^ are shown alongside local water column redox conditions (**b**). The bar chart plots the frequency that different biota are found in each redox zone^5^ (**a**). Large skeletal fossils and burrows are found exclusively in well-oxygenated settings and small skeletal fossils are largely restricted to well-oxygenated conditions, but may occur where conditions were only fleetingly oxic.

Samples were logged for fossil occurrences and sampled within established sequence stratigraphic frameworks, using detailed sedimentology^5^,^52^,^53^ (see Supplementary Notes 1 and 2). The presence of different forms of skeletal biota, soft-bodied biota and trace fossils are reported for precise horizons where geochemical analyses have been performed^5^, indicated by grey lines in Figs 2 and 3. General local ecology, supported by additional information from the literature, is also marked, without associated grey lines. Our sampling focused on carbonates and hence skeletal fossils are over-represented compared with soft-bodied biota and trace fossils. We define ‘large' skeletal animals as >10 mm in any dimension, which includes *Cloudina hartmannae*, some *Namacalathus* and *Namapoikia*.

### Rare earth elements in carbonate rocks

Rare earth elements and yttrium (REY) have a predictable distribution pattern in seawater and non-biological carbonate rocks should preserve local water column REY at the sediment–water interface^28^. Ce anomalies develop progressively, but cutoff values are established to define negative and positive anomalies. We define a negative anomaly as 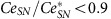, consistent with previous work^59^. A positive anomaly, using the same reference frame, would be defined as 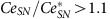. However, as positive anomalies are not previously described from carbonate sediments, we cautiously use a higher cutoff, 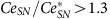, to ensure any positive anomalies are environmentally significant with respect to positive anomalies recorded from some modern manganous waters (1.21–2.43) (see Supplementary Note 3 and Supplementary Fig 11 for discussion of 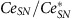 cutoffs). Although positive or negative Ce anomalies in carbonate rocks probably represent seawater redox conditions, the absence of any Ce anomaly (0.9–1.3) is somewhat equivocal and could result from anoxic water column conditions or overprinting of any Ce anomaly during diagenesis or leaching^31^. Alternately, Ce anomaly formation may be disrupted in surface waters because of wind-blown dust or photo-reduction of Mn oxides^60^. Fe (oxyhydr)oxides may also be REY carriers, but do not contain the clear Ce enrichments observed in Mn (oxyhydr)oxides (see Supplementary Note 4 for discussion of the role of Fe (oxyhydr)oxides in REY cycling).

Diagenetic phosphates, Fe and Mn (oxyhydr)oxides, organic matter and clays can potentially affect the REY signatures of authigenic sedimentary rocks if they are partially dissolved during the leaching process^61^,^62^,^63^. Care has been taken to partially leach samples, to isolate the carbonate phase without leaving excess acid, which may leach contaminant phases (see ref. 31 for detailed discussion of methodology). Powdered calcite samples were cleaned in Milli-Q water and pre-leached in 2% nitric acid, to remove adsorbed and easily exchangeable ions associated with clay minerals. The remaining sample was partially leached, also in 2% (w/v) nitric acid, to avoid contributions from contaminant phases such as oxides and clays^31^. The supernatant was removed from contact with the remaining residue, diluted with 2% nitric acid and analysed via inductively coupled plasma mass spectrometry in the Cross-Faculty Elemental Analysis Facility, University College London. This leaching method has been designed to extract the carbonate-bound REY pool without contributions from (oxyhydr)oxides or clays^31^. These same leachates were also analysed for major element concentrations (Mg, Fe, Mn, Al and Sr) via inductively coupled plasma optical emission spectrometry. Oxide interference was monitored using the formation rate of Ce oxide and the formation of 2+ ions was monitored using Ba^2+^. All REY concentrations were normalized to post-Archean Australian Shale.

Standard solutions analysed after every ten samples were within 5% of known concentrations. Replicate analyses on the inductively coupled plasma mass spectrometry give a relative s.d. <5% for most trace elements, with a larger s.d. for the heavy REE that sometimes have non-normalized concentrations <0.5 p.p.b. Carbonate standard material CRM 1c was prepared using the same leaching procedure as the samples and repeat analyses give a relative s.d. <5% for individual REY concentrations, and calculated Ce anomalies (average=0.80) give a relative s.d. <3%.

Mn/Sr ratios are <1 for the majority (97%) of samples and δ^18^Ocarb is >−10‰, indicating minimal open-system elemental and isotopic exchange during diagenesis, and excluding deep burial dolomitization (Supplementary Fig. 12). Ce anomaly data are only presented for carbonates that preserve seawater REY features (smooth patterns with molar Y/Ho>67)^28^,^31^, indicating they originate from the carbonate portion of the whole rock, without contributions from detrital or oxide phases. For samples with Y/Ho>67, 85% also have ∑REE <2 p.p.m. and all have ∑REE <10 p.p.m. La anomalies, and small positive Eu and Gd enrichments are prevalent in samples with Y/Ho>67 (Supplementary Fig. 11 and Supplementary Note 5 for discussion of Y anomaly thresholds). Positive Ce anomalies are associated with low Mn/Sr ratios (<1) and low Al, Zr, Ti, Fe and Mn contents in the leachate (<0.2 wt% for Fe and <500 p.p.m. for Mn), indicating minimal contamination due to diagenetic exchange, leaching of clays or Fe–Mn (oxyhydr)oxide phases (Supplementary Fig. 12).

### Rare earth elements in shales

Shales from throughout the Zebra River section, including inter-reef deposits and lateral subordinate shales between grainstone horizons, were fully digested using HNO_3_-HF-B(OH)_3_-HClO_4_ at the University of Leeds. These full digestions include the dominant siliciclastic component, but would also encompass any subordinate (oxyhydr)oxide phases, organic matter or carbonate components. The full digestions were dried down, washed twice in 50% nitric acid and resuspended in 2% nitric acid for analysis on an inductively coupled plasma mass spectrometry in the Cross-Faculty Elemental Analysis Facility, University College London.

Relative to standardized shale composition, post-Archean Australian Shale^64^, the Zebra River shales show consistent patterns (Supplementary Fig. 13), with middle-REY enrichment (bell-shaped index=1.25) and negative Y anomalies (shale-normalized Y/Ho=0.88), but no anomalous Ce behaviour. These patterns resemble those reported for Fe (oxyhydr)oxides^65^,^66^ and may well derive in part from the high Fe_ox_ contents of these shales (up to 1.2%). Shales carry a ‘continental-type' REY pattern and represent a baseline from which surface-solution fractionation of REY begins, and thus they are commonly used to normalize seawater REY patterns.

### Fe speciation in carbonates and siliciclastics

The Fe speciation method quantifies Fe that is (bio)geochemically available in surficial environments (termed Fe_HR_) relative to Fe_T_. Mobilization and subsequent precipitation of Fe in anoxic water column settings results in Fe_HR_ enrichments in the underlying sediment. The nature of anoxia (that is, sulfide-rich or Fe-containing) is determined by the extent of sulfidation of the Fe_HR_ pool^1^. Fe speciation data for carbonate rock samples discussed here and accompanying interbedded siliciclastic rocks come from previously published data^5^. The Fe-speciation technique was performed using well-established sequential extraction schemes^1^,^67^. The method targets operationally defined Fe pools, including carbonate-associated-Fe (Fe_Carb_), ferric oxides (Fe_Ox_), magnetite (Fe_Mag_), pyrite Fe (Fe_Py_) and Fe_T_. Fe_HR_ is defined as the sum of Fe_carb_ (extracted with Na-acetate at pH 4.5 and 50 °C for 48 h), Fe_ox_ (extracted via Na-dithionite at pH 4.8 for 2 h), Fe_mag_ (extracted with ammonium oxalate for 6 h) and Fe_py_ (calculated from the mass of sulfide extracted during CrCl_2_ distillation). Fe_T_ extractions were performed on ashed samples (8 h at 550 °C) using HNO_3_-HF-H_3_BO_3_-HClO_4_. All Fe concentrations were measured via atomic absorption spectrometry and replicate extractions gave a relative s.d. of <4% for all steps, leading to <8% for calculated Fe_HR_. Fe_Py_ was calculated from the wt% of sulfide extracted as Ag_2_S using hot Cr(II)Cl_2_ distillation^68^. A boiling HCl distillation before the Cr(II)Cl_2_ distillation ruled out the potential presence of acid volatile sulfides in our samples. Pyrite extractions give reproducibility for Fe_py_ of 0.005 wt%, confirming high precision for this method. Analysis of a certified reference material (PACS-2, Fe_T_=4.09±0.07 wt%, *n*=4; certified value=4.09±0.06 wt%) confirms that our method is accurate. Replicate analyses (*n*=6) gave a precision of ±0.06 wt% for Fe_T_ and a relative s.d. of <5% for the Fe_HR_/Fe_T_ ratio.

Calibration in modern and ancient marine environments suggests that Fe_HR_/Fe_T_<0.22 indicates deposition under oxic water column conditions, whereas Fe_HR_/Fe_T_>0.38 indicates anoxic conditions^1^. Ratios between 0.22–0.38 are considered equivocal and may represent either oxic or anoxic depositional conditions. For sediments identified as anoxic, Fe_py_/Fe_HR_>0.8 is diagnostic for euxinic conditions and Fe_py_/Fe_HR_<0.7 defines ferruginous deposition^1^. Although originally calibrated for siliciclastics^1^,^32^, enrichments in Fe_HR_/Fe_T_ can also be identified in carbonates deposited under anoxic water column conditions^69^. These Fe_HR_ enrichments can far exceed Fe_HR_ contents expected under normal oxic deposition, where trace amounts (∼0.1 wt%) of Fe may be incorporated into carbonates, or precipitate as Fe–Mn coatings^69^. However, although early dolomitization in shallow burial environments does not generally cause a significant increase in Fe_HR_, late-stage deep-burial dolomitization may significantly increase Fe_HR_^69^, but there is no petrographic evidence for deep-burial dolomitization in our samples^5^,^16^. Consistent with a recent calibration^69^, we have limited the application of Fe speciation to carbonate samples with >0.5 wt% Fe_T_, which buffers against the impact of non-depositional enrichments in Fe_HR_^69^. Where Fe_T_ is very low (<0.5wt%), this may indicate deposition under oxic conditions^69^. In addition, however, we stress that all of our redox interpretations based on Fe speciation in carbonates are entirely consistent with data from siliciclastic horizons interbedded with and/or associated with carbonate rocks contained within the same m- to dm-scale depositional cycle^5^.

Equivocal Fe_HR_/Fe_T_ ratios could be a consequence of dilution of a high water column Fe_HR_ flux through rapid sedimentation^32^ (for example, in turbidite settings) or post-depositional transformation of unsulfidized Fe_HR_ minerals to less reactive sheet silicate minerals^8^,^67^. Further, local Fe_HR_ enrichments can occur due to preferential trapping of Fe_HR_ in inner shore or shallow marine environments (for example, flood plains, salt marshes, deltas and lagoons). However, none of the presented Fe_HR_ data here are from rocks that show evidence of turbiditic deposition and are from dominantly open marine settings. Oxidative weathering may result in mineralogical transformation of Fe minerals. Oxidation of siderite would transfer Fe_carb_ to the Fe_ox_ pool and hence any interpretation of ferruginous or euxinic signals would remain robust. The weathering of pyrite to Fe_ox_ would not affect interpretation of anoxic signals (Fe_HR_/Fe_T_>0.38), but may reduce the Fe_py_/Fe_HR_ ratio, giving a false ferruginous signal in a euxinic sample. In the extreme and highly unlikely scenario that all Fe_ox_ in our samples is a product of pyrite weathering, ∼10% of the anoxic samples would give a euxinic signal. However, significant Fe_carb_ (>20% of the Fe_HR_ fraction) occurs in ∼57% of anoxic samples, indicating that the rocks have not been completely weathered and hence this extreme scenario is unlikely.

### Data availability

All relevant data are available to download in the data repository associated with this manuscript and further details on the Fe-speciation data are available in ref. 5.

## Additional information

**How to cite this article:** Tostevin, R. *et al.* Low-oxygen waters limited habitable space for early animals. *Nat. Commun.* 7:12818 doi: 10.1038/ncomms12818 (2016).

## Supplementary Material

Supplementary Information Supplementary Figures 1 - 13, Supplementary Table 1, Supplementary Notes 1 - 5, Supplementary Discussion and Supplementary References

## Figures and Tables

**Table 1:** Framework for co-interptetation of Ce_SN_/Ce_SN_* and Fe-speciation data on the same samples.

	**Ce anomaly**		
**Fe-speciation**	**Negative anomaly**	**Equivocal (no anomaly)**	**Positive anomaly**
Anoxic			
Fe_HR_/Fe_T_>0.38, Fe_py_/Fe_HR_<0.7	NA	Ferruginous	Ferruginous
			
Equivocal			
Fe_HR_/Fe_T_ 0.22–0.38	Oxic	Unknown	Manganous
			
Oxic			
Fe_HR_/Fe_T_<0.22, Fe_T_<0.5wt% (likely to be oxic)	Oxic	Oxic	Manganous

Fe_HR_, highly reactive Fe; Fe_T_, total Fe; NA, not applicable.
