# Supplementary material for: Low-oxygen waters limited habitable space for early animals
Source: Nat Commun. 2016 Sep 23;7:12818. doi: 10.1038/ncomms12818 (PMC5036156; doi:10.1038/ncomms12818)
Supplement: Supplementary Information — Supplementary Figures 1 - 13, Supplementary Table 1, Supplementary Notes 1 - 5, Supplementary Discussion and Supplementary References [file ncomms12818-s1.pdf]

Supplementary Figures

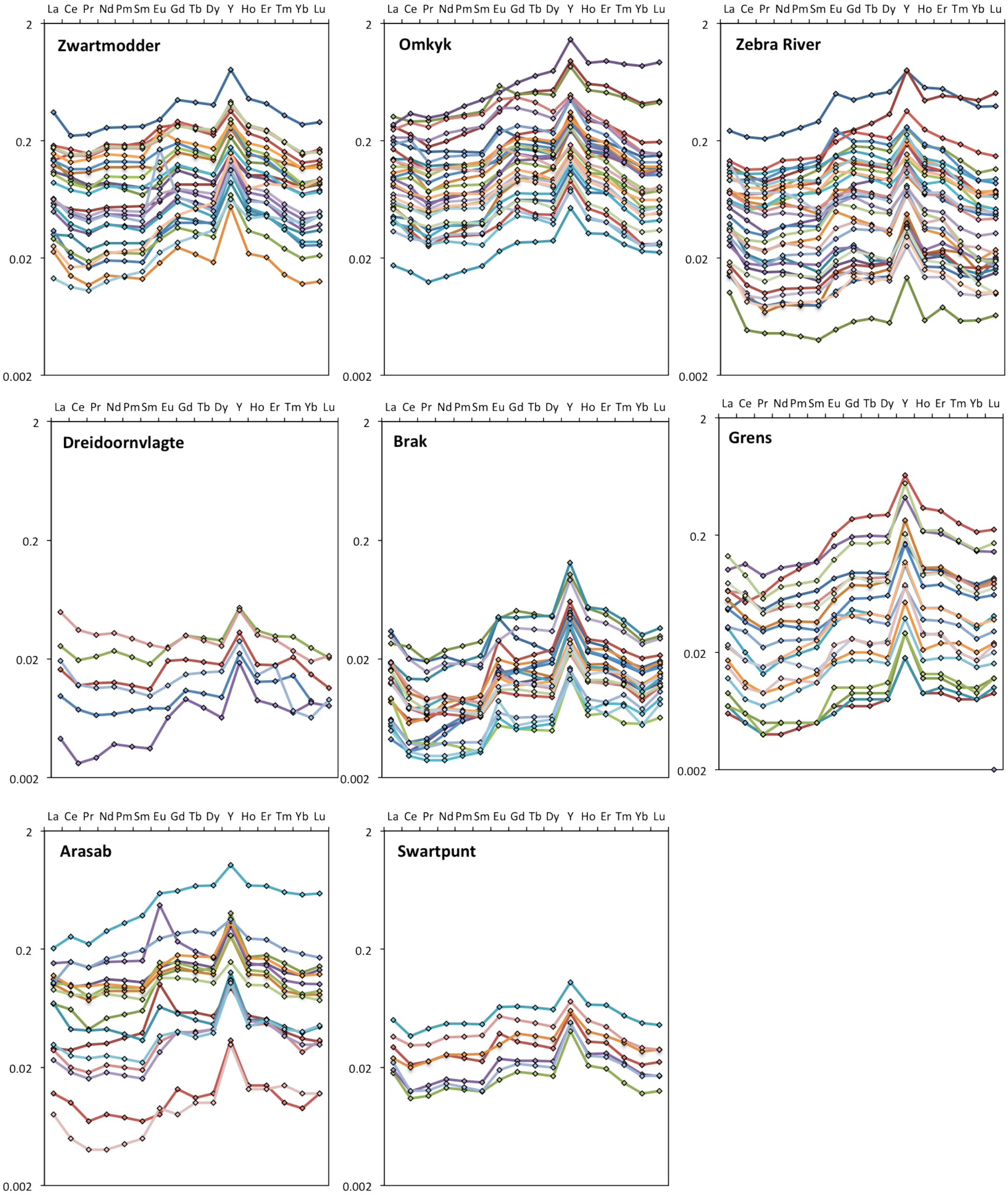

Supplementary Figure 1: REY spider diagrams for each locality, screened for samples with Y/Ho>67.

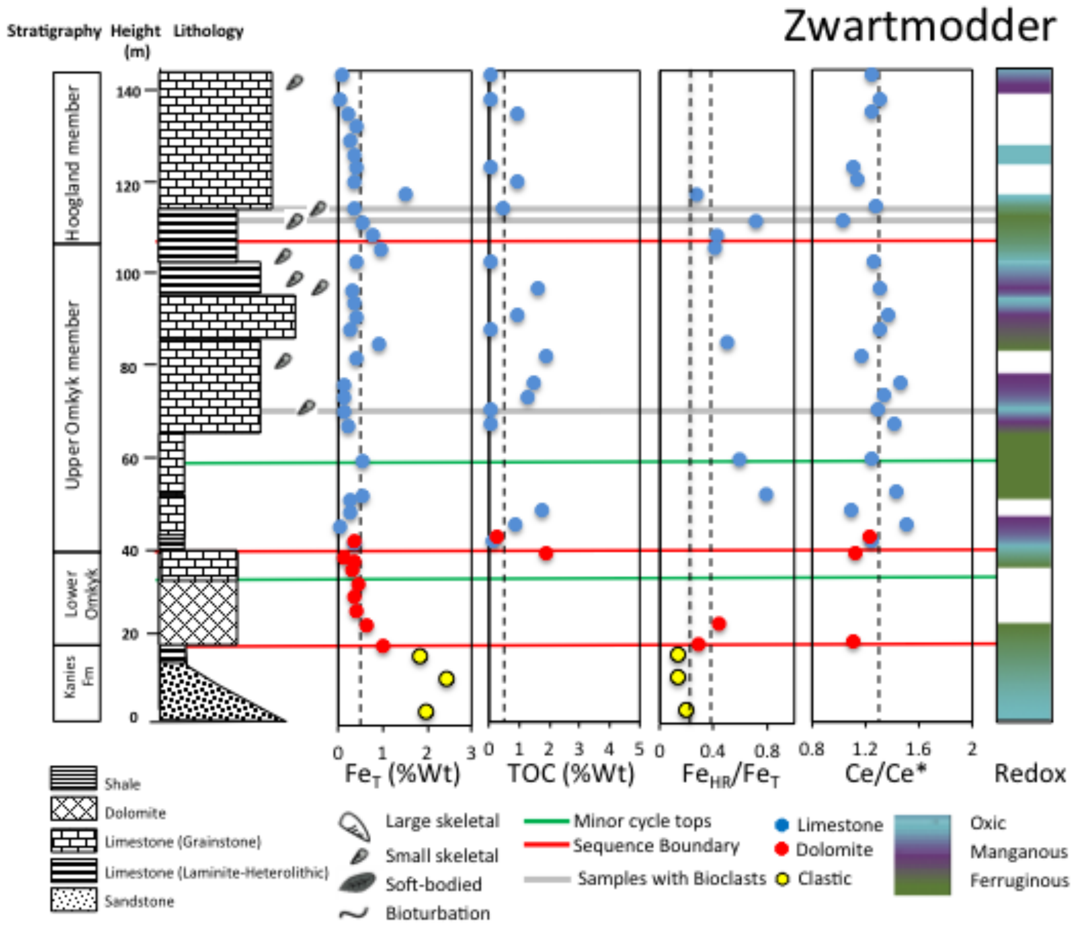

Supplementary Figure 2: Stratigraphic and data summary for Zwartmodder

Fe_T, Fe_{HR}/Fe_T, TOC[2] and Ce_{SN}/Ce^{*}_{SN} data for inner ramp Zwartmodder from the Kuibis Subgroup in the Zaris Basin, alongside stratigraphic log[2]. The final column represents our best interpretation of redox based on all these data.

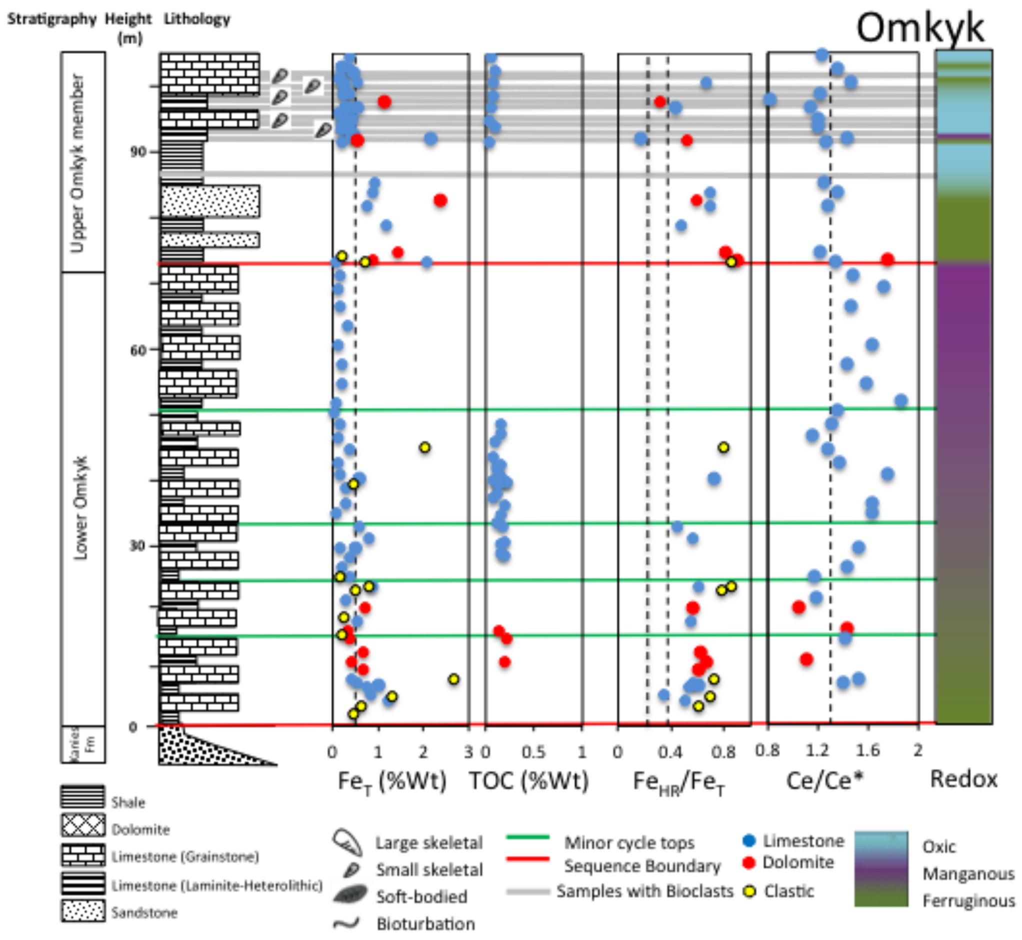

Supplementary Figure 3: Stratigraphic and data summary for Omkyk

Fe_T, Fe_{HR}/Fe_T, TOC[2] and Ce_{SN}/Ce^*_{SN} data for deep inner ramp Omkyk from the Kuibis Subgroup in the Zaris Basin, alongside stratigraphic log[2]. The final column represents our best interpretation of redox based on all these data.

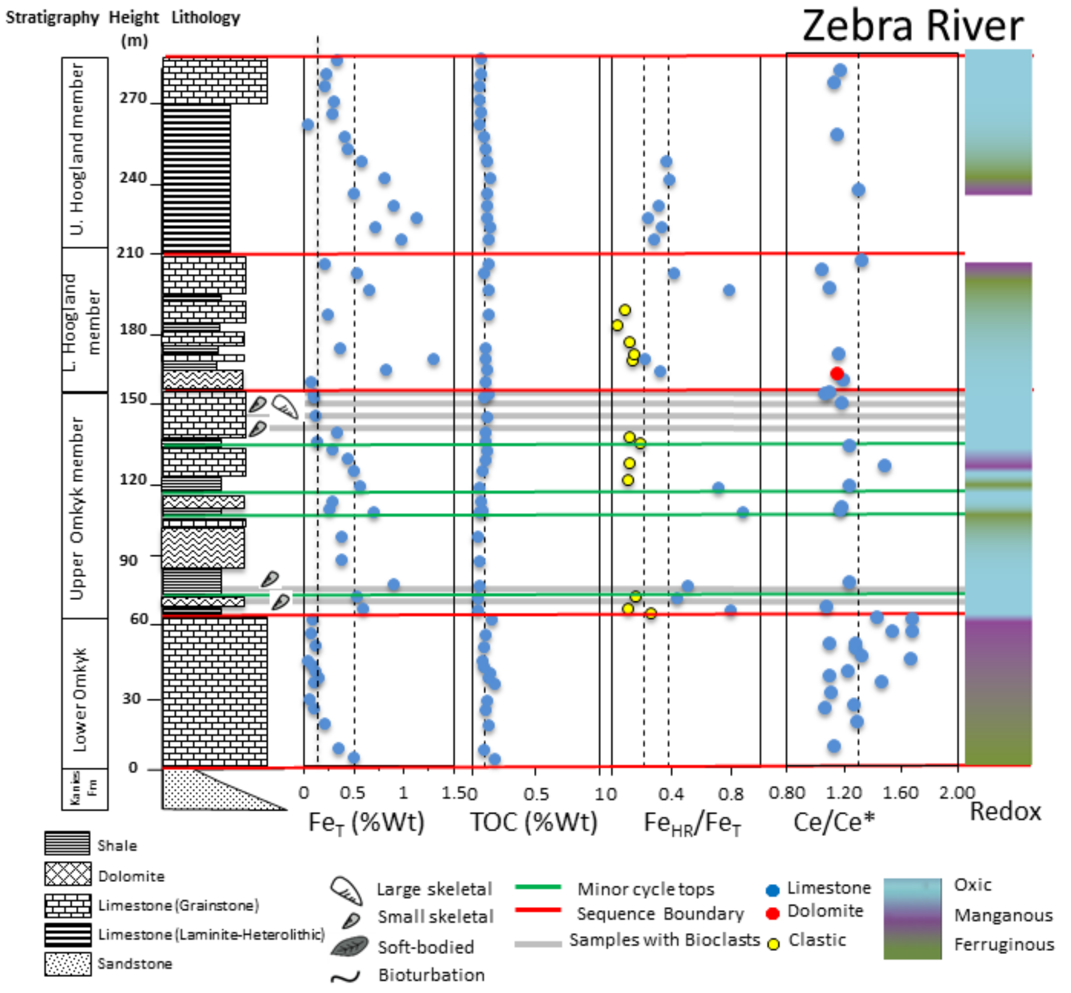

Supplementary Figure 4: Stratigraphic and data summary for Zebra River

Fe_T, Fe_{HR}/Fe_T, TOC[2] and Ce_{SN}/Ce^*_{SN} data for deep inner ramp Zebra River from the Kuibis Subgroup in the Zaris Basin, alongside stratigraphic log[2]. The final column represents our best interpretation of redox based on all these data.

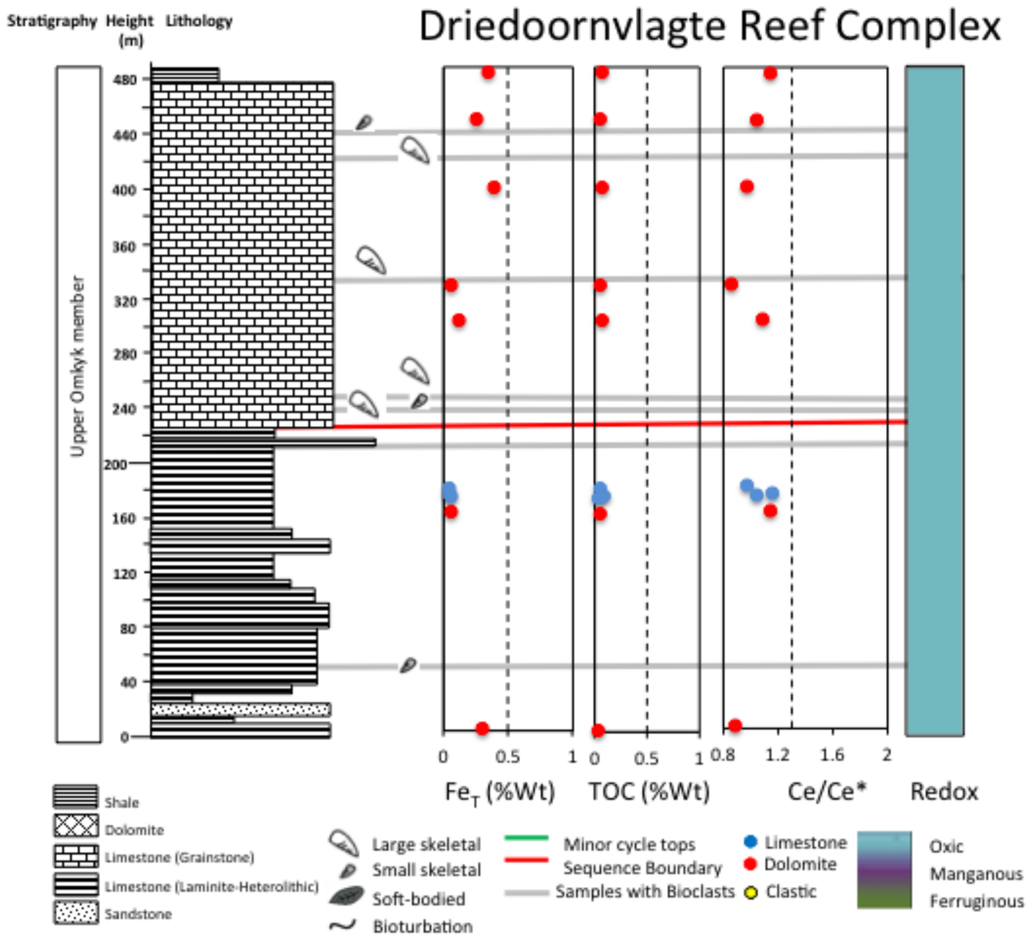

Supplementary Figure 5: Stratigraphic and data summary for Dreidoornvlagte

Fe_T, Fe_{HR}/Fe_T, TOC[2] and Ce_{SN}/Ce^*_{SN} data for mid-ramp pinnacle reef Driedoornvlagte from the Kuibis Subgroup in the Zaris Basin, alongside stratigraphic log.[2] The final column represents our best interpretation of redox based on all these data.

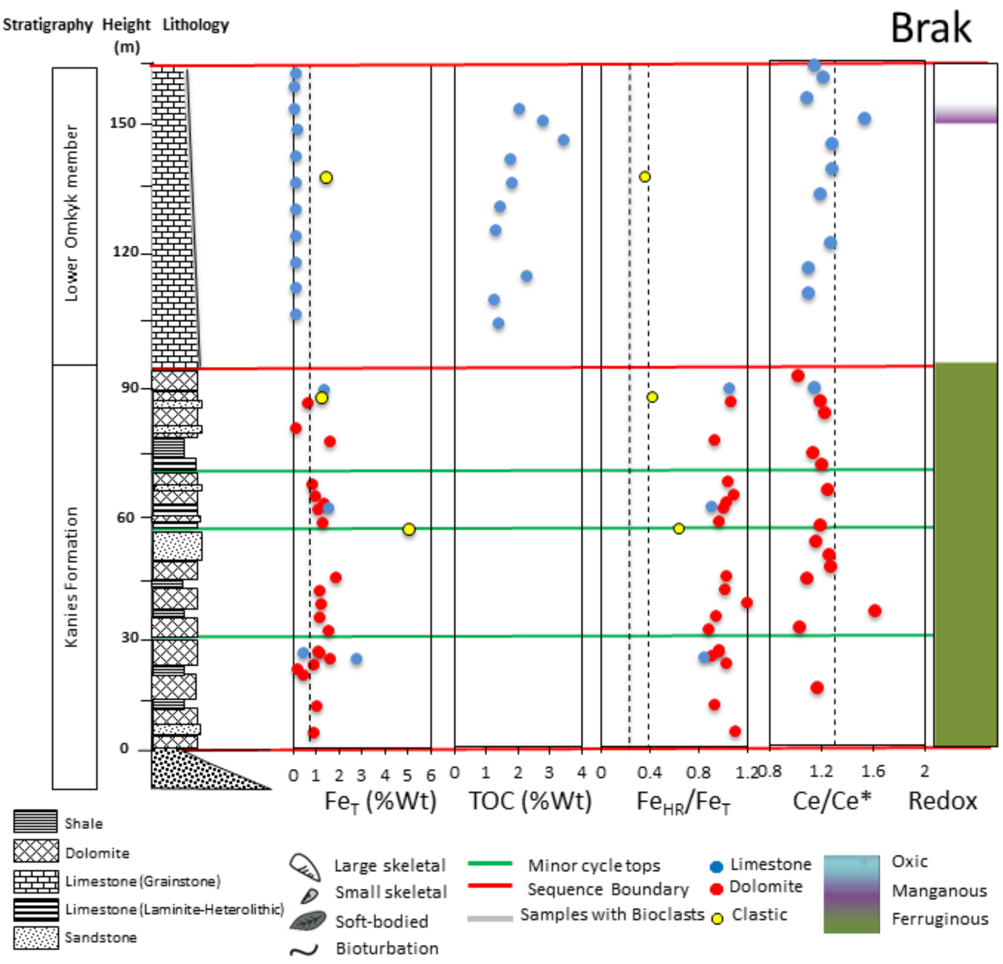

Supplementary Figure 6: Stratigraphic and data summary for Brak

Fe_T, Fe_{HR}/Fe_T, TOC[2] and Ce_{SN}/Ce^*_{SN} data for outer ramp Brak from the Kuibis Subgroup in the Zaris Basin, alongside stratigraphic log[2]. The final column represents our best interpretation of redox based on all these data.

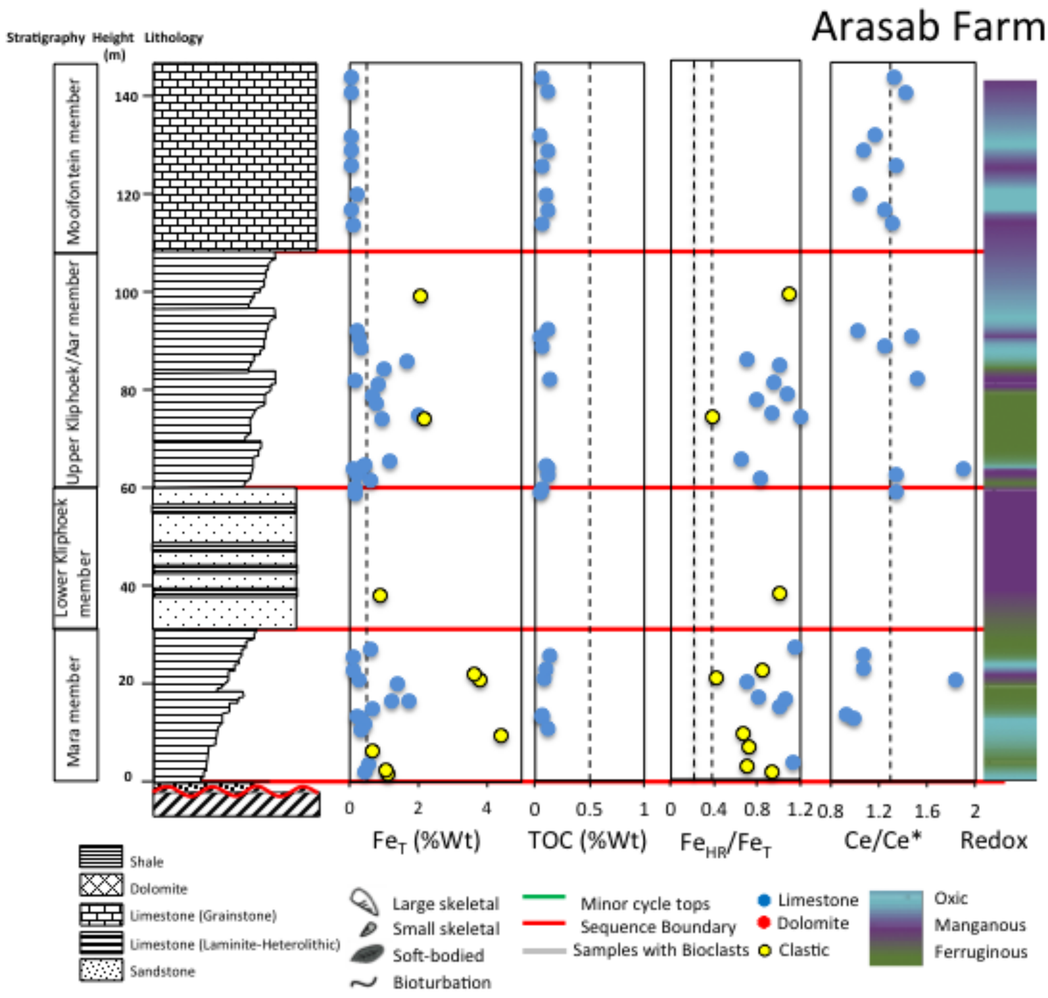

Supplementary Figure 7: Stratigraphic and data summary for Arasab

Fe_T, Fe_{HR}/Fe_T, TOC[2] and Ce_{SN}/Ce^*_{SN} data for inner ramp Arasab from the Kuibis Subgroup in the Witputs Basin, alongside stratigraphic log[2]. The final column represents our best interpretation of redox based on all these data.

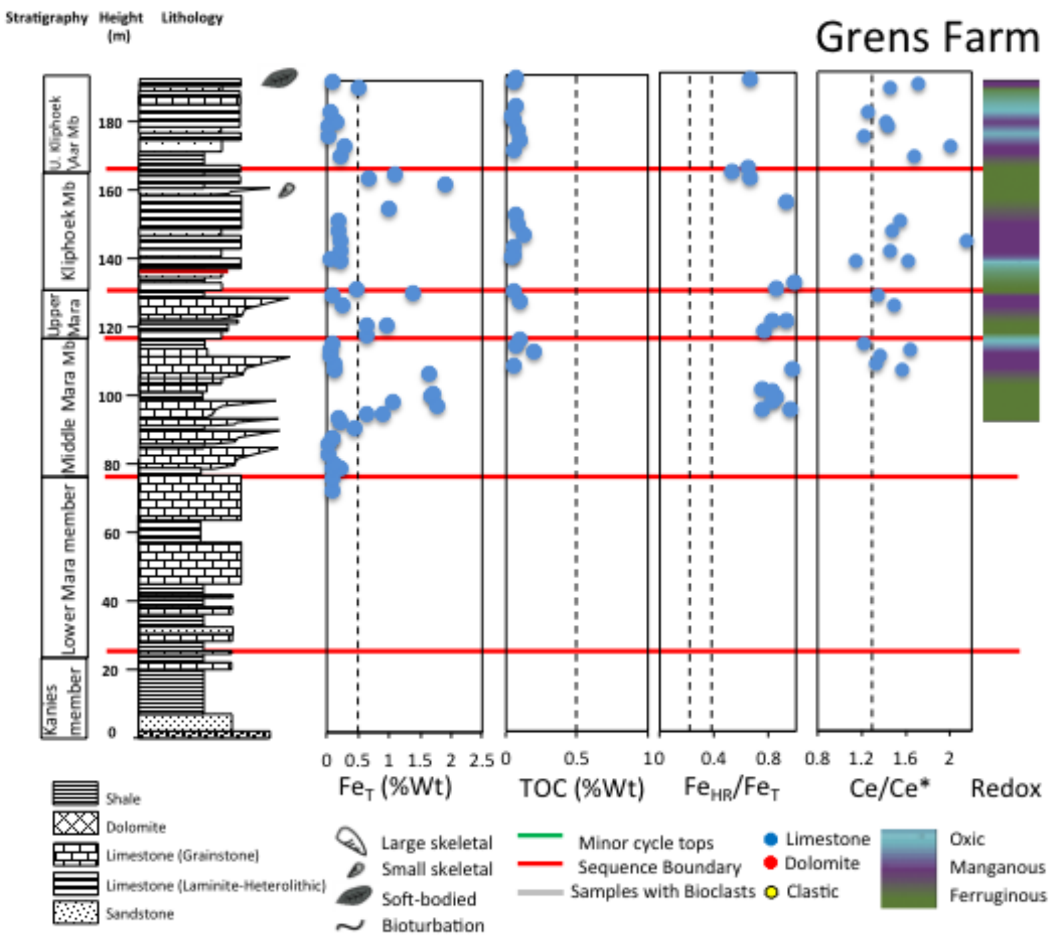

Supplementary Figure 8: Stratigraphic and data summary for Grens

Fe_T, Fe_{HR}/Fe_T, TOC and Ce_{SN}/Ce^*_{SN} data for inner ramp Grens from the Kuibis Subgroup in the Witputs Basin, alongside stratigraphic log[2]. The final column represents our best interpretation of redox based on all these data.

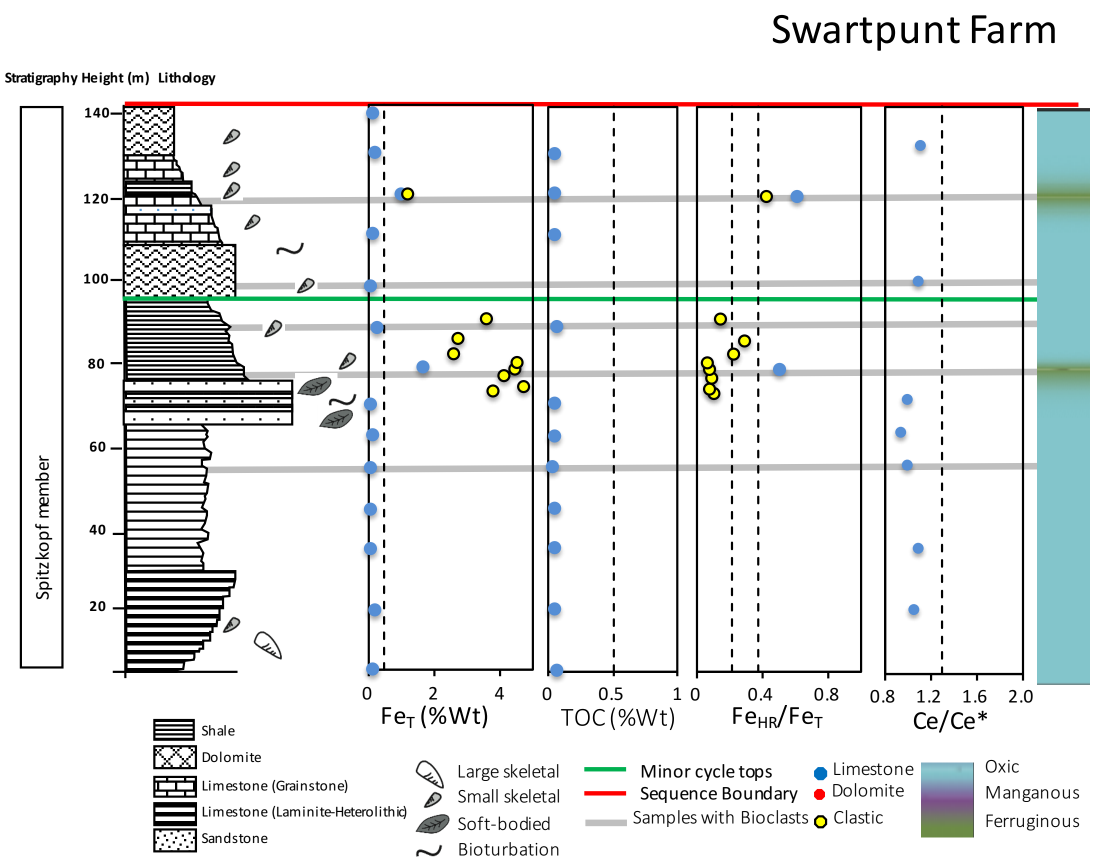

Supplementary Figure 9: Stratigraphic and data summary for Swartpunt

Fe_T, Fe_{HR}/Fe_T, TOC[2] and Ce_{SN}/Ce^*_{SN} data for inner ramp Swartpunt from the Schwarzrand Subgroup in the Witputs Basin, alongside stratigraphic log[2]. The final column represents our best interpretation of redox based on all these data.

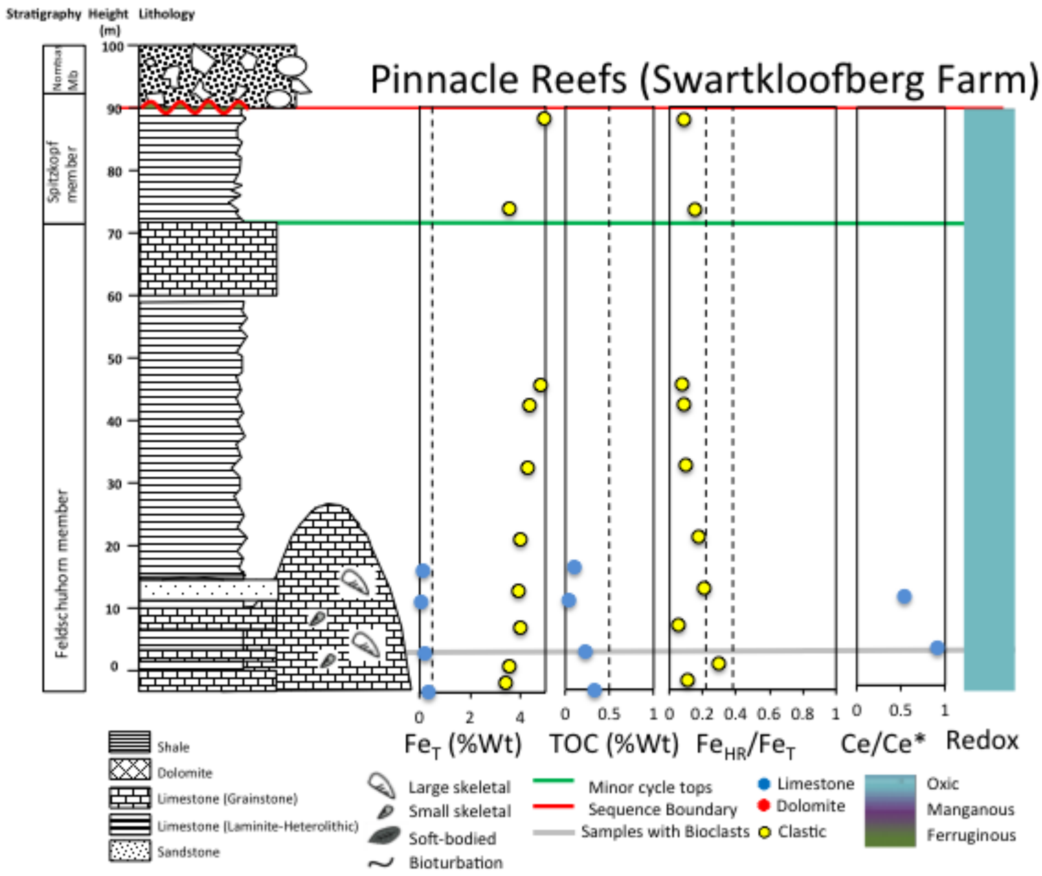

Supplementary Figure 10: Stratigraphic and data summary for the Pinnacle Reefs

Fe_T, Fe_{HR}/Fe_T, TOC[2] and Ce_{SN}/Ce^*_{SN} data for inner ramp Pinnacle reef from the Schwarzrand Subgroup in the Witputs Basin, alongside stratigraphic log[2]. The final column represents our best interpretation of redox based on all these data.

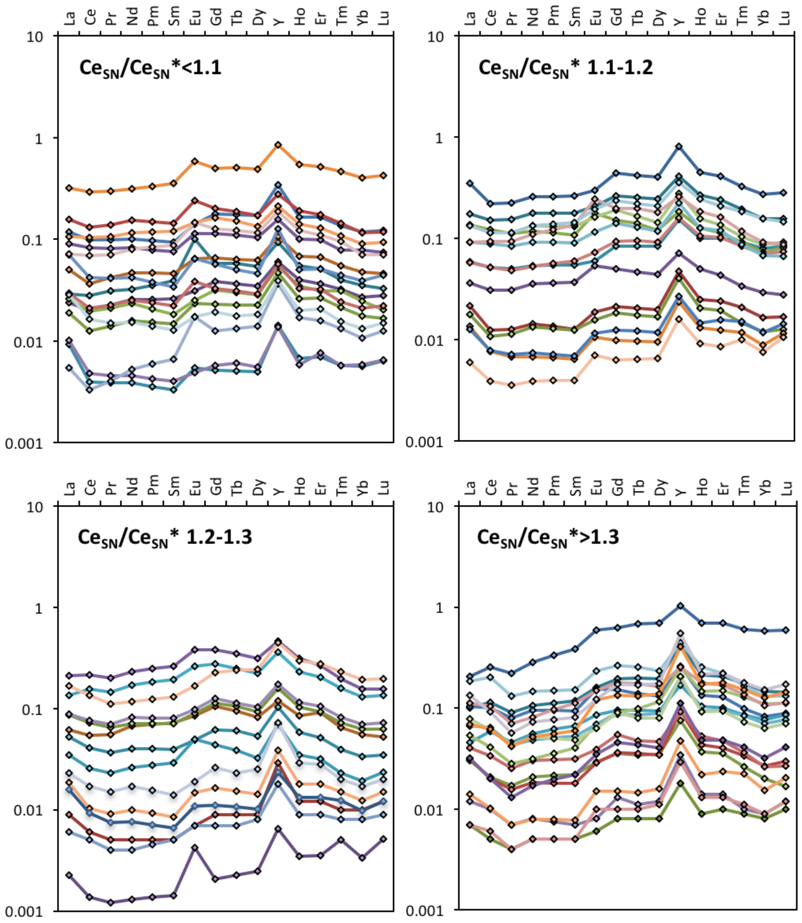

Supplementary Figure 11: **Comparison of REY spider diagrams for examples with no Ce anomaly (<1.3), and those with a positive Ce anomaly (>1.3).**

Both show seawater features, including positive Y and La anomalies, and some samples show small positive Eu anomalies. REY patterns for samples with no positive Ce anomaly are divided into categories <1.1, 1.1-1.2 and 1.2—1.3, to demonstrate the utility of 1.3 as an appropriate cut off for genuine Ce enrichments above background fluctuations.

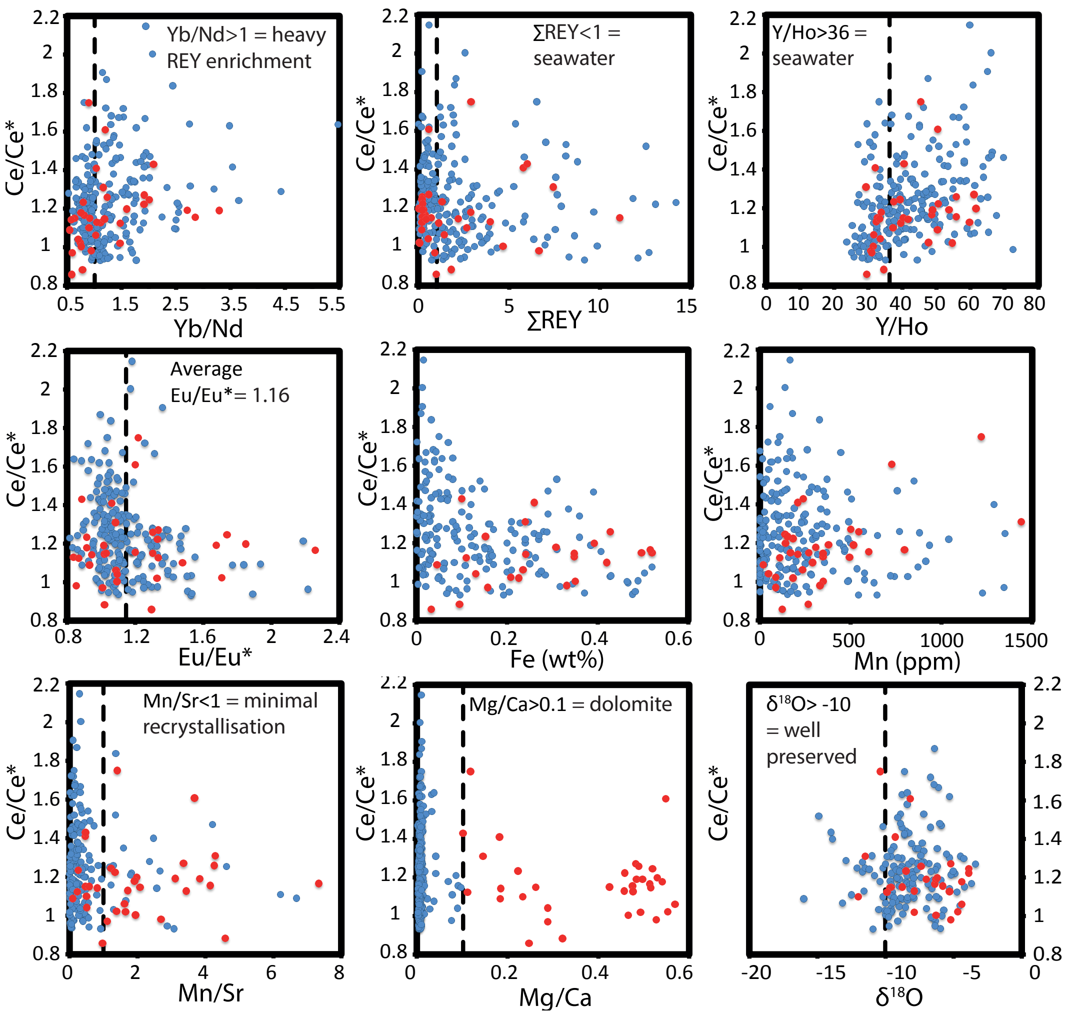

Supplementary Figure 12: Positive Ce anomalies against common REY, major element and isotopic parameters for assessing data quality.

Key threshold values highlighted by dashed lines. Red = dolomite, blue = limestone.

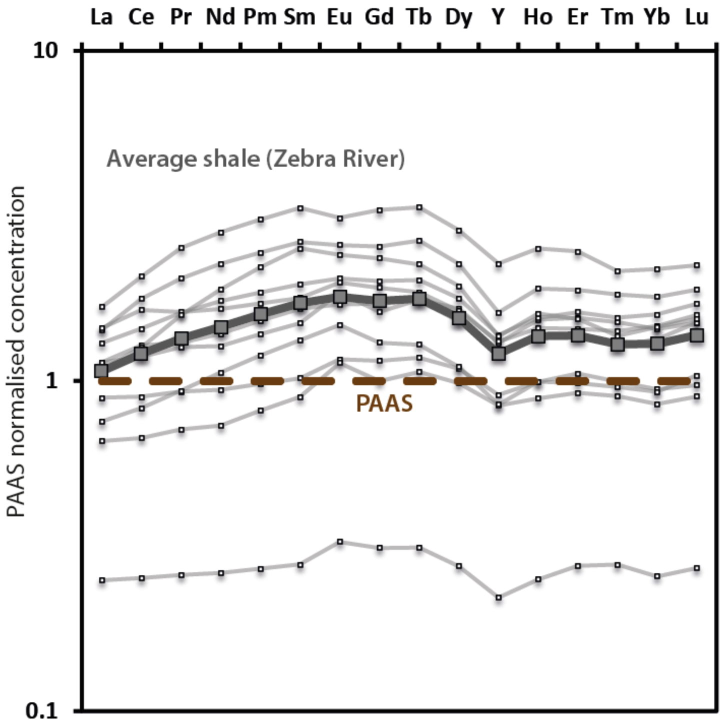

Supplementary Figure 13: Rare earth elements for shales from Zebra River section.

Data are normalised to PAAS, and show small negative Y anomalies and minor MREE enrichment, but no anomalous Ce behaviour. Thick grey line represent average shale values for Zebra River, and brown dashed line represents international shale standard, PAAS[1].

Supplementary Tables

Supplementary Table 1. Co-ordinates and names and Site numbers for all sampled sections.

Relative water depths are indicated, assessed based on sedimentology[2]

Section	Longitude	Latitude	Relative water depth
Site 1: Zwartmodder	16°19'31.00"E	24°53'41.00"S	Shallow inner ramp
Site 2: Omkyk	16°13'45.00"E	24°48'19.00"S	Deep inner ramp
Site 3: Zebra River	16°12'13.24" E	24°30' 49.38"S	Deep inner ramp
Site 4: Driedornvlagte	16°39' 50.57"E	23°51' 36.83"S	Mid ramp
Site 5: Brak	16°8'6.50"E	23°58'17.00"S	Outer ramp
Site 6: Arasab	16°24'47.00"E	26°53'15.00"S	Shallow inner ramp
Site 7: Grens	16°21'58.50"E	27°10'34.50"S	Shallow inner ramp
Site 8: Pinnacle Reefs	16°3’40.60"E	27°27’52.70"S	Mid ramp
Site 9: Swartpunt	16°41'33.00"E	27°28'29.00"S	Shallow inner ramp

Supplementary Notes

Supplementary Note 1 - Sequence Stratigraphy

The Zaris and Witputs Basins have distinct stratigraphic frameworks[3,4], but common features such as systems tracts and sequence boundaries can be correlated across the Osis Arch, which separates the two sub-basins[5,6]. The basal unit in both basins is the siliciclastic Kanies Member. In the Zaris Basin, this is overlain by thick carbonate dominated units of the Omkyk and Hoogland Members. The Omkyk member is typified by decimeter-scale, upwards coarsening, mid-inner to shoreface cycles. The overlying Hoogland Member is a deeper water mid-inner ramp to middle shoreface settings, recording transgressive-regressive depositional cycles in heterolithic interbeds. In the Witputs Basin, the Kanies Member is overlain by the carbonate dominated Mara Member deposited during a highstand systems tract. The Mara Member is overlain by the transgressive systems tract of the Kliphoek Member, typified by upper shoreface to tide-dominated shoreline sandstones[3]. The overlying Mooifontein Member is a thin-bedded shallow-marine limestone, the top of which is marked by a conglomerate-filled unconformity with canyon-like erosional relief[4].

The younger Schwarzrand Group, sampled in the Witputs Basin, records mostly shallow marine mixed clastic and carbonate rocks in the lower Urusis Formation. The middle part consists of carbonate dominated Huns, Feldschuhorn and Spitzkopf Member, including pinnacle reefs that initiate on the flooding surface at the top of the Huns Member and are enveloped by the overlying shales of the

Feldschuhorn Member. The top of the Schwarzrand Group is the conglomeratic Nomstas Formation, that infills incised valleys, bracketed by ash beds dated at 542.68 ± 1.245 Ma and 540.61 ± 0.67 Ma (U–Pb zircon)[7].

Typical late Ediacaran fossils, such as *Ernietta, Pteridinium, Swartpuntia,* and *Rangea*[8], vendotaenids[9], and Ediacaran skeletal fossils, *Cloudina*[5,10], *Namacalathus*[11] and *Namapoikia*[12], have been described from both the Zaris and Witputs Basins[2]. The oldest reported *Cloudina* is from the carbonate-dominated Mara Member in the Witputs Basin[5]. The oldest Ediacara-type fossils occur in the Mara Member of the Kuibis Subgroup in the Witputs Basin[3], and the youngest, including *Swartpuntia*, are found 60 m below the Ediacaran–Cambrian unconformity in the more southerly portion of the Witputs Basin[8].

Supplementary Note 2 – Section and data descriptions

Zwartmodder was deposited predominantly in shallow, proximal ramp settings. The section is dominated by dolomites at the base, shallowing to mainly limestone laminates, packstones and grainstones towards the top of the section. There are horizons of abundant skeletal animals in the Upper Omkyk Member highstand systems tract (HST) and Lower Hoogland Member transgressive systems tract (TST), and minor thrombolites in the Upper Omkyk Member HST[2]. There is at least one horizon with large skeletal fossils. Zwartmodder shows fluctuating anoxia, with Fe_{HR}/Fe_T >0.38 interbedded with carbonates with low Fe_T[2]. Other samples with low Fe_T have high TOC, giving an equivocal signal. Both Fe_T and TOC are variable, from 0 to ~2 wt% and 0.04 to 5.66wt%, respectively

(Supplementary Fig 5). Intervals where Fe_T is <0.5 wt% are intermittent in the Upper Omkyk Member TST and HST, and Lower Hoogland Member TST, and are interpreted to represent probable oxic horizons. Fe speciation in shales in the Kanies at the base of the section indicates an oxic signature. Limited carbonate samples with FeT > 0.5 wt% indicate intermittently anoxic ferruginous conditions in the Lower Omkyk Member TST and Upper Omkyk Member TST and HST, and Lower Hoogland Member TST. Positive Ce anomalies are prevalent but do not reach values as high as in deeper inner-ramp sections [range: 1.04–1.51, average: 1.26±0.13 (1 s.d.)] (Supplementary Fig 5). Across all samples the average Y anomaly =75.0, BSI =1.2, Eu/Eu* =1.1 Yb_{SN}/Nd_{SN} =1.1, and ∑REE =2.68 ppm.

At deep inner-ramp Omkyk, the lower part of the succession is dominated by slope turbidites, slumps and storm-beds and then shallows in the upper part to limestone grainstones showing tidal influence, together with limited thrombolites and abundant skeletal animals. Some shoaling cycles contain evidence for deposition in supra- to inter-tidal conditions, which may have been subjected to exposure and evaporitic conditions. There are horizons of abundant skeletal animals in the Upper Omkyk Member late TST only[2]. Omkyk shows very similar stratigraphic patterns to Zebra River (Supplementary Fig 7), with some stable periods of oxygenated conditions towards the top of both the Lower and Upper Omkyk Member. Fe_T is highly variable, from 0.02 to 2.66 wt%. (Supplementary Fig 6). Carbonate intervals where Fe_T is <0.5 wt% are intermittent in the Lower and Upper Omkyk Member TST and HST, and are interpreted to represent probable oxic horizons. Fe speciation in carbonates and

shales throughout the lower part of the Lower Omkyk Member, and the Upper Omkyk Member indicates anoxic ferruginous conditions. Positive Ce anomalies increase up to the top of the Lower Omkyk Member [range: 1.04-1.87, average: 1.39±0.22 (1 s.d.)], suggesting these conditions were manganous, rather than well-oxygenated. In the Upper Omkyk Member, Ce anomalies are close to 1 [range: 0.83-1.75, average: 1.28±0.20 (1 s.d.)]. There is one carbonate sample, containing biota, that is identified as 'oxic' via Fe-speciation (Fe_{HR}/Fe_T <0.22) [Ref 2], and is associated with a positive Ce anomaly, indicating manganous low oxygen conditions. Another sample containing biota is associated with a negative Ce anomaly indicating well-oxygenated conditions. One sample containing skeletal biota is associated with an anoxic ferruginous signal (Fe_{HR}/Fe_T >0.38), but the remaining biota-bearing samples are associated with low Fe_T and an equivocal Ce_{SN}/Ce_{SN}^{*} close to 1. Across all samples the average Y anomaly =68.4, BSI =1.2, Eu/Eu* =1.1 Yb_{SN}/Nd_{SN} =1.3, and ∑REE =5.5 ppm.

At the deep inner-ramp locality, Zebra River, the Lower Omkyk Member is dominated by grainstones. In the TST of the Upper Omkyk Member, thrombolite-stromatolite reefs nucleate, forming laterally continuous biostrome layers. *Cloudina* and *Namacalathus* can be found within thrombolite heads and lag beds within inter-reef shales. Towards the top of the Upper Omkyk Member the section shallows into grainstone dominated facies with subordinate shale horizons, containing thinner, discontinuous biostrome microbial reef systems, and some large Namacalathus <35 mm^2. Trace fossils are reported from sandstone lenses at nearby Donkergange Farm[13]. The Hoogland Member contains storm dominated laminites and heterolithics, shallowing towards

grainstone-dominated facies. At Zebra River, Fe-speciation identifies persistent oxygenation, with anoxic ferruginous conditions confined to short-lived horizons[2] (Supplementary Fig 7). Fe_T in carbonate rocks is highly variable, from 0.03 to 1.28 wt%, and follows sequence stratigraphic changes with elevated Fe_T at the base of sequences during early TSTs. Carbonate intervals where Fe_T is <0.5 wt% are present throughout the Lower Omkyk Member, the late TST of the Upper Omkyk Member, the late TST of the Lower Hoogland Member, and the entire Upper Hoogland Member, and are interpreted to represent probable oxic horizons. Fe-speciation on shales and carbonate rocks indicates oxic conditions in the HST of the Upper Omkyk Member, and in the TST of the Upper Omkyk Member and the lower Hoogland member. Anoxic ferruginous conditions are limited to the early TST of the Upper Omkyk Member and the Lower Hoogland Member, and the HST of the Upper Omkyk Member. Large positive Ce_{SN}/Ce_{SN}^{*} anomalies [range: 1.06-1.68, average: 1.34±0.21 (1 s.d.)] are observed towards the top of the Lower Omkyk Member in grainstone units (Supplementary Fig 7). Some intermittent, smaller positive anomalies are present towards the top of the Upper Omkyk Member, but the majority of samples show Ce_{SN}/Ce_{SN}^{*} close to 1 [range: 1.01-1.48, average: 1.18±0.10 (1 s.d.)]. Across all samples the average Y anomaly =71.0, BSI =1.2, Eu/Eu* =1.1 Yb_{SN}/Nd_{SN} =1.1, and ∑REE =2.41ppm.

Driedoornvlagte reef accumulated in a mid-ramp setting, during the TST of the Upper Omkyk Member. The build-up is 7 km wide and a total of 200 m thick: this thickness reflects the incised accommodation space associated with the deeper water setting compared to Zebra River reefs. The build-up is predominantly composed of thrombolitic limestones and grainstones, with

minor dolomitisation. Skeletal animal fossils are abundant, particularly towards the top of the section where *Cloudina* and large *Namapoikia* (up to a meter) are found associated with thrombolites[2,12]. *Namacalathus* is also abundant lower in the section. Large, reef-building *Cloudina hartmannae* (up to 8 mm tube diameter, several cm length) and fissure-inhabiting *Namapoikia* are found towards the top of the section[12,14,15]. Driedoornvlagte has consistently low Fe_T, ranging from 0.03 to 0.07%, and low TOC, ranging from 0.005 to 0.383 wt% (Supplementary Fig 8). This is interpreted to indicate persistently oxic conditions[2]. No bioturbation has been noted, but preservation may be poor in such energetic settings. Bar two negative anomalies (0.88 and 0.86), Driedoornvlagte exhibits Ce_{SN}/Ce^{*}_{SN} close to unity throughout [range: 0.86-1.14, average: 1.03±0.10 (1 s.d.)] (Supplementary Fig 8). The two negative Ce anomalies are present in samples with superchondritic Y/Ho ratios, but below the >67 threshold we have assigned to screen for clay leaching. We suggest these samples, with higher ∑REE, have been affected by partial leaching of clays, and so the true magnitude of the negative Ce anomalies is likely higher. We include the data in Fig 1 as negative Ce anomalies cannot result from clay or oxide leaching, or alteration, and so likely record original seawater redox conditions [16]. Across all samples the average Y anomaly =75.8, BSI =1.1, Eu/Eu* =1.1 Yb_{SN}/Nd_{SN} =0.96, and ∑REE =0.57ppm.

Brak is a deep water section, which is dominated by fine-grained dolostones inter-bedded with thin sandstones, shales and quartzites indicative of deeper, outer ramp conditions, with shallow subtidal grainstones (limestones) present only towards the very top of the section. There are no animal fossils or

thrombolites present, but some stromatolites occur at approximately 30 m in the Kanies Member[2]. Fe_T is variable, from 0.05 to ~1.89 wt% in the Kanies Member, and from 0.08 to 0.132 in the limestones of the Lower Omkyk Member. Fe-speciation indicates anoxic and ferruginous conditions throughout all carbonates and shales in the Kanies member at Brak[2]. No unequivocal redox proxy data are available for the Lower Omkyk Member as all Fe_T < 0.5 wt%, but TOC is notably >0.5 wt%, ranging from 1.22 to 3.39 wt% (Supplementary Fig 9). Ce_{SN}/Ce^*_{SN} is close to 1.2 throughout, consistent with anoxic conditions, with one notable positive Ce anomaly [range: 0.967-1.530, average: 1.18±0.12 (1 s.d.)] (Supplementary Fig 9). The Y anomaly is notably high in most samples from Brak, average =94.1. The BSI is low =1.1, Yb_{SN}/Nd_{SN} ratios are high (average=1.8), and ∑REE is low (average =0.2 ppm). The Europium anomalies are the largest observed across the basin (range 1.02 - 2.25, average=1.5).

Arasab was deposited in shallow, proximal ramp settings. The base of the section is predominantly composed of limestone units (often displaying evaporitic fabrics) punctuated by thin shale interbeds, transitioning to thick quartzite beds of the Lower Kliphoek Member, followed by interbedded shale and limestone of the Upper Kliphoek Member. The section is capped by a thick limestone unit of the Mooifontein Member, containing distinct oolite bands in the lower reaches. Though the Lower Kliphoek quartzite and Aar Member units are known to contain abundant soft-bodied animal fossils in other localities[17], none have been noted at Arasab[2]. Arasab shows fluctuating anoxia, with anoxic signals in carbonates with high Fe_T interbedded with carbonates with low Fe_T (likely oxic)[2]. Fe_T data from shales range from 0.51 to 0.77wt% and Fe speciation data

indicate fully anoxic ferruginous conditions. Carbonate rocks in the Mara, Kliphoek, and Aar Members have Fe_T > 0.5 wt%, likewise indicating ferruginous conditions. In samples with Fe_T < 0.5 wt%, TOC is low, ranging from 0.05 to 0.13 wt%; these samples are from rocks interbedded with anoxic layers in the Mara and Aar Members, and continuously through the Mooifontein Member, and are taken to indicate sporadic short-lived intervals of probable oxic water column conditions although no bioturbation has been noted. In this locality, REY were only analysed in samples with Fe_T <0.5 wt%, to maximise the proportion of samples that give a non-contaminated signal[16]. Positive Ce anomalies are common, [range: 0.93–1.91, average: 1.28±0.26 (1 s.d.)] with positive anomalies most prevalent in the Upper Kliphoek (Aar) Member, and largely absent in the Mara and Mooifontein Members (Supplementary Fig 10). Several samples show large positive Eu anomalies. Across all samples the average Y anomaly =97.5, BSI =1.2, Eu/Eu* =1.0 Yb_{SN}/Nd_{SN} =1.5, and ∑REE =2.1 ppm.

Grens includes all members of the Dabies Formation of the Kuibis Subgroup. Conglomerates, sandstones and shales dominate the lowermost 20 m, defining the Kanies Member at Grens, with overlying facies of the Mara Member predominantly composed of limestone with shale inter-beds clearly defining patterns of shoaling cyclicity (further evidence from evaporitic fabrics). The Kliphoek Member is composed of limestone with small layers of quartzite, and the capping Aar Member consists of shale, limestone, and dolo-cemented quartzite. At 180 m in-situ assemblages of the Ediacaran fossil *Nemiana* are abundant in coarse sandstone layers[2], for which redox data is not available. Fe_T data was >0.5 wt% for only 17 of the 50 samples analysed, with Fe_{HR}/Fe_T for

these 17 samples indicating anoxic ferruginous conditions. Where Fe_T is <0.5 wt%, TOC is very low, from 0.04 to 0.2 wt%. This is taken as an indication of probable oxic conditions although no bioturbation has been noted, and these horizons are found interbedded with anoxic horizons in the upper Middle and Kliphoek Members. Grens shows positive Ce anomalies as high as 2.15, with highest values in the Kliphoek and Aar Member (Supplementary Fig 11) [range: 1.14–2.15, average: 1.50±0.25 (1 s.d.)]. Only samples with <0.5 wt% Fe_T have been analysed for REY in this section. Across all samples the average Y anomaly =104.6, BSI =1.1, Eu/Eu* =1.1 Yb_{SN}/Nd_{SN} =1.9, and ∑REE =0.9 ppm.

Swartpunt, from the younger Schwarzrand Subgroup, extends close to the Neoproterozoic-Cambrian Boundary and transitions from high-energy inner ramp conditions at the base, followed by a TST, and then shallowing to inner ramp carbonates at the top of the section. At the base of the section, laminated limestone dominates, containing *Namacalathus,* large *Cloudina* and other bioclastic material at some levels. Carbonate microbialites are also found at this level, some with a 'leopardskin' texture. Overlying the limestones are beds of upwards coarsening of green mudstone and to ripple-laminated and thick bedded planar-laminated or cross-bedded sandstones, containing burrows and soft-bodied fossils including *Swartpuntia* and *Pteridinium*[8]. These in turn give way to more thin-bedded limestones and dolomites with dm-scale thrombolites, and thick- and thin-bedded limestones with rare flat-pebble and intraclastic breccias deposited in shallow to sub-tidal ramp settings below storm wave base. These in turn give way to a HST of laminated, flaggy limestones containing small (<5 mm) *Cloudina riemkeae* and thrombolites. Horizontal burrows are first noted

~60 m below the Ediacaran-Cambrian unconformity[18]. All but two of the Fe_T values from Swartpunt carbonates are <0.5 wt%, and these also show consistently low TOC throughout, from 0.04 to 0.07wt% (Supplementary Fig 12). This is taken to indicate oxic conditions, and is confirmed by the presence of complex bioturbation in the upper 60m of the section. TST clastic samples consistently contain Fe_T > 0.5 wt%. Fe_{HR}/Fe_T values vary significantly through the section, but all clastics show an oxic signature. By contrast, two HST carbonate samples with Fe_T > 0.5 wt% occurring some 55 m and 20 m below the Ediacaran-Cambrian unconformity show Fe_{HR} /Fe_T > 0.38, with low Fe_{py}/Fe_{HR} giving an anoxic ferruginous signature. Overall, Fe-speciation suggests persistent oxygenation with two limited anoxic ferruginous horizons[2,19]. Swartpunt carbonates show no positive Ce anomalies (Supplementary Fig 12), which is consistent with well-oxygenated conditions throughout most of Swartpunt. Across all samples the average Y anomaly =69.4, BSI =1.2, Eu/Eu* =1.3, Yb_{SN}/Nd_{SN} =1.1, and ∑REE =2.5 ppm.

The Pinnacle Reefs at Swartkloofberg Farm, southern Namibia, were deposited within TSTs in a mid-ramp setting. They initiated on the flooded surface of the Huns Platform. At the base of the reefs, reef carbonate inter-fingers with siltstones and shales, but the reefs then grew up to 40 m topographic relief above the sea floor. After termination of reef growth, they were enveloped by the shales of the Feldschuhorn Member, of which they now form a part. Mixed communities of skeletal animals of varied sizes persist at the Pinnacle Reefs[2]. The sampled reef was composed predominantly of microbialite, with some laminar micritic layers. Microbialite textures are predominantly stromatolitic at the base (approximately 0–

5m), with thrombolitic textures appearing at approximately 5 m height, and aggregations of Namacalathus (up to 12 mm diameter) becoming more prevalent towards the top of the reef. Carbonate samples from within one of the reefs have low Fe_T with values ranging from 0.042-0.33 wt%, and low TOC, with values ranging from 0.02-0.04 wt%. This is interpreted to indicate oxic conditions. Siliciclastics from nearby Felschuhorn Member (bar one) have Fe_{HR}/Fe_T <0.22, suggesting persistently oxygenated conditions. ∑REE is low for the four carbonates (0.07-0.39), meaning non-normalised HREE concentrations fell below the detection limit. This precludes interpretation of Y anomalies, but La, Ce, Pr and Nd are recorded with sufficient resolution to interpret Ce anomalies for two samples. One negative Ce anomaly (0.53) is recorded, and one other sample shows no anomaly (Supplementary Fig 13).

Supplementary Note 3 – Ce_{SN}/Ce^*_{SN} cut off values

Cut-off values used to define negative Ce anomalies are well established (<0.9), but here we report positive Ce anomalies, which have rarely been described from modern or ancient carbonate rocks. We assign a cut off value of >1.3. This is firstly, because we are cautious about interpreting Ce enrichments that are not significant with respect to those observed in modern manganous waters. Secondly, fluctuations in Pr and Nd concentrations, encompassing natural variation and precision during ICP-MS analysis (accurate to within 5%), may result in a calculated Ce anomaly up to 1.2. Because of this, REY patterns need to be screened by eye to show that genuinely anomalous Ce enrichments, relative to the trajectory from the MREE to the LREE, are evident in all samples with

Ce_{SN}/Ce^{*}_{SN} >1.3 (Supplementary Fig 3). All of our patterns have been additionaly screened to select true Ce enrichments above baseline fluctuations and trends.

We have considered the effect on our data and interpretation of applying a different cut-off, e.g. 1.1 or 1.2. Using a cut-off of >1.3, the total number of samples identified as well-oxygenated was 191; low oxygen, 58; and anoxic, 141. Using a threshold of >1.2 results in 30 samples categorised as oxic switching to manganous, and threshold of >1.1 results in an additional 34 samples switching from oxic to manganous. Some of these environments host small skeletal fossils, and so the total number of small shelly fossils in low oxygen environments would also increase. The pinnacle reefs and Driedoornvlagte, which both foster large skeletal animals and complex, long-lived communities, remain persistently oxic regardless of the cut-off used. We emphasise that where Ce_{SN}/Ce^{*}_{SN} is between 1.1 and 1.3, no genuine Ce enrichment is observed in REY patterns, and so there is no indication that these samples represent manganous environments (Supplementary Fig 3).

Supplementary note 4 – The role of Fe in Ce cycling

The REY are considered to be scavenged quantitatively onto Fe (oxyhydr)oxides, and fractionated during scavenging onto Mn (oxyhydr)oxides. This is supported by evidence from hydrothermal plumes[20], water column data[21–25], marine Fe-Mn nodules[26], Fe-formation REY data[27] and experimental work[28–31]. However, there is conflicting evidence from the sequential leaching of Fe-Mn nodules that suggests Fe (oxyhydr)oxides may be important in controlling REY fractionation[32,33]. Fe (oxyhydr)oxides may scavenge the middle-REE

preferentially, and exclude Y, but there is only limited evidence that they preferentially accumulate Ce[31,34]. The relative importance of Fe and Mn in controlling Ce oxidation and scavenging is debated, but Mn is generally considered more important for controlling the redox cycling of Ce[20–29].

We assume that Mn (oxyhydr)oxides are the primary carrier for REY in the oceans. If Fe(oxyhydr)oxides were important carriers of Ce, we would anticipate positive Ce anomalies to co-occur with Fe enrichments, but they co-occur with Mn enrichments. Nevertheless, we consider how our interpretation of the data would be affected in the case that Fe (oxyhydr)oxides were important Ce carriers. Firstly, highly reactive Fe enrichments occur in fully anoxic conditions, and so there would be an apparent discrepancy between very low Fe_T and Ce enrichments in the same sample. However, Ce(IV) may undergo reductive dissolution and be released from the surface of Fe (oxyhydr)oxides at a Ce redoxcline independently of other REY. In this case, the carrier phase becomes unimportant, as Ce reduction and release from the surface would occur independently of either Mn or Fe reduction. Further, there is some evidence that Ce(IV) forms a discrete solid oxide phase[34], in which case Ce cycling would occur independently of both Mn and Fe cycling, and at higher oxygen concentrations. However, most evidence supports that Ce fractionation can only occur on the surface of solid Mn (oxyhydr)oxides[33], and therefore tracks Mn redox cycles.

In the Nama Group, we suggest that Mn (oxyhydr)oxides were the main carrier for REY and that the release of excess Ce occurred under manganous conditions. Support for Mn (oxyhydr)oxides as the main carrier phases comes from the

decoupling of Fe and Mn alongside positive Ce anomalies. Mn concentrations may be considerably affected by diagenesis, and so Mn concentration data should be interpreted with caution. Although Mn/Fe ratios are relatively elevated in manganous samples, absolute Mn concentrations and Mn/Sr ratios remain low, consistent with unaltered carbonate.

Supplementary note 5 - Yttrium anomaly thresholds

Positive Ce anomalies occur in samples with seawater features, and similar REY patterns to samples without positive Ce anomalies (Supplementary Fig 3). Although a molar Y/Ho ratio >67 is traditionally used to screen for seawater REY signals[35–37], the interpretation of Y anomalies in our samples is not straightforward, as the dissolution of Mn (oxyhydr)oxides beneath a redoxcline may alter the full REY pattern along with release of excess Ce. Mn (oxyhydr)oxides contain a negative Y anomaly when normalized to ambient waters, but this is not as pronounced as the negative Y anomaly in co-occurring Fe (oxyhydr)oxides[33]. Excess Ce may accumulate beneath a redoxcline via two mechanisms: reduction of Ce(IV) and release from the oxide surface, or reductive dissolution of the Mn (oxyhydr)oxide and release of all oxide bound REY. In the former case, Ce would be enriched in the water without affecting Y. In the latter, redox dissolution may impart a small effect on the Y anomaly. Since the development of Y anomalies is controlled by scavenging onto particles across all depths, including onto Fe (oxyhydr)oxides that would remain intact in a partially oxic manganous zone, excess Y would likely be ubiquitous in the ocean (see data from modern manganous zones with positive Y and Ce anomalies in Fig 1). Further, suspended (oxyhydr)oxide particles in the Rainbow hydrothermal

plumes and ancient Fe-Formations[20,38] exhibit superchondritic Y anomalies, despite their residence time in the water column exceeding experimental constraints on Y equilibration times[28]. This suggests that Mn (oxyhydr)oxides may retain the superchondritic Y anomaly of ambient waters, particularly during rapid precipitation, and yet continue to accumulate Ce in oxic waters. The magnitude of Y anomalies in our samples is controlled by scavenging, reductive dissolution of (oxyhydr)oxides in seawater and any partial clay leaching during acid dissolution[16]. Although the magnitude of the Y anomaly may reduce slightly in seawater in the manganous zone, we do not attempt to interpret the absolute magnitude of Y/Ho, but employ a cut off >67 to exclude samples affected by partial leaching of clay minerals

Supplementary Discussion

Alternative mechanisms for Ce enrichment

Anomalous local REY input

Although REY inputs from rivers or wind blown dust generally carry a flat REY signature[39,40], if terrestrial input was anomalous in the Ediacaran this could explain Ce enrichment in the basin. However, the lack of anomalous Ce behavior in local shales (Supplementary Fig 1 and methods) indicates that the positive Ce anomalies recorded in carbonates cannot be attributed to unusual local REY inputs. Moreover, if positive Ce anomalies resulted from surface maxima[23] we would expect them to be most prevalent in inner ramp sections (Zwartmodder, Arasab and Swartpunt). Although limited Ce enrichments are recorded in these sections, they are more pronounced in deeper inner ramp sections (Zebra River, Omkyk, Grens).

Alkaline waters

Highly alkaline waters in unusual lake settings may exhibit positive Ce anomalies due to stabilization of Ce complexes in the water column[41,42]. There is no evidence that the Nama Group was highly alkaline, and carbon and strontium isotope data both support the Nama Group being normal marine and fully connected to the open ocean[2,43].

Ce oxidation in the presence of siderophores

The presence of Fe-chelating siderophores, such as desferrioxamine B (DFOB), can affect the mobilization of REY during the weathering of igneous rocks [44,45]. This process may affect the input of REY into the basin from terrestrial weathering, but we find no anomalous Ce behavior in terrestrial input (see Methods and Supplementary Fig 1). Further, under Fe-limiting conditions in the water column, microbes release siderophores to scavenge iron from Fe(III) mineral phases, and since Ce-DFOB complexes are orders or magnitude more stable than Ce-carbonate complexes, water column Ce enrichments may occur in unusual environments where siderophore activity is prevalent[46]. However, where bio-available Fe(II) is present in the water column, it is unlikely that siderophores were common. In addition, the presence of siderophores results in enriched Ce alongside extensive La depletion[46], which is in marked contrast to the positive La anomalies observed in the Nama Group.

Reductive dissolution of Fe-Mn (oxyhydr)oxides during diagenesis.

Although seawater REY in carbonate rocks are thought to be preserved during early diagenesis[37], REY released from Fe-Mn (oxyhydr)oxide dissolution in anoxic pore waters could potentially be captured during recrystallization from aragonite to calcite. Modern pore water data rarely show anomalous Ce enrichments[34,47,48], even though Mn (oxyhydr)oxides presumably carry positive Ce anomalies into the sediment[33]. This suggests that there is an additional REY carrier that dominates the REY content of pore waters. One possible candidate is organic matter[34,49], but the REY signal of organic matter is poorly understood, and the low TOC concentrations in most of our samples suggest this was not an important contributor[2]. A more likely candidate is the dissolution of Fe (oxyhydr)oxides.

Pore waters often become fully anoxic within millimeters of the sediment-water interface, and so Mn and Fe (oxyhydr)oxides commonly undergo simultaneous dissolution, or form discrete zones but separated on a cm-scale[50]. If the REY, Fe and Mn in our samples were derived entirely from pore water resetting, the excess Fe over Mn present in all samples would reflect higher Fe contents in the pore waters, as both elements have a partition coefficient close to one [Ref [51]]. Fe (oxyhydr)oxides usually carry an MREE-enriched signal with a negative Y anomaly (similar to shales presented here, Supplementary Fig 1), and this signal would dominate anoxic pore waters[34,52], but this REY pattern is not observed in our carbonate leach, even where Fe concentrations in carbonate are high. This argues against destruction of seawater REY patterns via incorporation of pore water REY. However, in the water column, Mn (oxyhydr)oxide dissolution can occur in the absence of Fe (oxyhydr)oxide dissolution, in a stable meter -

decimeter thick manganous zone. A water column origin for carbonate-bound REY is the only way to account for the relatively enriched Mn/Fe ratios, but with higher absolute Fe concentrations, and positive Ce anomalies alongside superchondritic Y anomalies.

REY concentrations in pore waters are an order of magnitude higher than in seawater, and the REY content of carbonates should increase during diagenetic exchange, alongside increases in Fe and Mn contents. But positive Ce anomalies correlate with lower ∑REE, [Fe] and [Mn] (Supplementary Fig 2). Because positive Ce anomalies do not correlate with diagenetic proxies (low $\delta^{18}O_{carb}$, high Mn/Sr), or with proxies for other forms of potential contamination (low Y/Ho, high Fe or Mn concentrations etc.), but instead with stratigraphic trends, we can rule out wholesale diagenetic exchange of REY as a primary control.

Diffusion from reducing inshore sediments into seawater

Mobilization of Ce from reducing inshore sediments[53] may explain local Ce peaks, but Ce is rapidly oxidized in overlying oxic waters. The positive Ce reported here occurs in sediments with low TOC and oxic Fe-speciation signals[2]. If excess Ce originates from diffusion above reducing seafloor sediments, we would expect the Ce anomalies to peak in anoxic waters, but instead, they are observed predominantly in oxic waters.

Supplementary References

1. Pourmand, A., Dauphas, N. & Ireland, T. J. A novel extraction chromatography and MC-ICP-MS technique for rapid analysis of REE, Sc and Y: Revising CI-chondrite and Post-Archean Australian Shale (PAAS) abundances. *Chemical Geology* **291,** 38–54 (2012).
2. Wood, R. A. *et al.* Dynamic redox conditions control late Ediacaran ecosystems in the Nama Group, Namibia. *Precambrian Research* **261,** 252–271 (2015).
3. Saylor, B. Z., Grotzinger, J. P. & Germs, G. J. B. Sequence stratigraphy and sedimentology of the Neoproterozoic Kuibis and Schwarzrand Subgroups (Nama Group), southwestern Namibia. *Precambrian Research* **73,** 153–171 (1995).
4. Saylor, B. Z., Kaufman, A. J., Grotzinger, J. P. & Urban, F. A Composite Reference Section for Terminal Proterozoic Strata of Southern Namibia. *SEPM Journal of Sedimentary Research* **68,** (1998).
5. Germs, G. J. B. Implications of a sedimentary faceis and depositional environmental analysis of the Nama group in South West Africa/Namibia. *Geological Society of South Africa* **11,** 89–114 (1983).
6. Germs, G. J. B. The Neoproterozoic of southwestern Africa, with emphasis on platform stratigraphy and paleontology. *Precambrian Research* **73,** 137–151 (1995).
7. Grotzinger, J. P., Bowring, S. A., Saylor, B. Z. & Kaufman, A. J. Biostratigraphic and Geochronologic Constraints on Early Animal Evolution. *Science* **270,** 598 –604 (1995).

8. Narbonne, G. M., Saylor, B. Z. & Grotzinger, J. P. The youngest Ediacaran fossils from southern Africa. *Journal of Paleontology* **71.06,** 953–967 (1997).
9. Germs, G. J., Knoll, A. H. & Vidal, G. Latest Proterozoic microfossils from the Nama Group, Namibia (South West Africa). *Precambrian Research* **32,** 45–62 (1986).
10. Grant, S. W. Shell structure and distribution of Cloudina, a potential index fossil for the terminal Proterozoic. *Am J Sci* **290-A,** 261–294 (1990).
11. Grotzinger, J. P., Watters, W. A. & Knoll, A. H. Calcified metazoans in thrombolite-stromatolite reefs of the terminal Proterozoic Nama Group, Namibia. *Paleobiology* **26,** 334 –359 (2000).
12. Wood, R. A., Grotzinger, J. P. & Dickson, J. A. D. Proterozoic Modular Biomineralized Metazoan from the Nama Group, Namibia. *Science* **296,** 2383 –2386 (2002).
13. Macdonald, F. A., Pruss, S. B. & Strauss, J. V. Trace fossils with spreiten from the late Ediacaran Nama Group,Namibia: complex feeding patterns five million years before the Precambrian–Cambrian boundary. *Journal of Paleontology* **88,** 299–308 (2014).
14. Penny, A. *et al.* Ediacaran metazoan reefs from the Nama Group, Namibia. *Science* **344,** 1504–1506 (2014).
15. Wood, R. & Curtis, A. Extensive metazoan reefs from the Ediacaran Nama Group, Namibia: the rise of benthic suspension feeding. *Geobiology* **13,** 112–122 (2015).
16. Tostevin, R. *et al.* Effective use of cerium anomalies as a redox proxy in carbonate-dominated marine settings. *Chemical Geology* **438,** 146–162 (2016).

17. Hall, M. *et al.* Stratigraphy, palaeontology and geochemistry of the late Neoproterozoic Aar Member, southwest Namibia: Reflecting environmental controls on Ediacara fossil preservation during the terminal Proterozoic in African Gondwana. *Precambrian Research* **238,** 214–232 (2013).
18. Jensen, S. & Runnegar, B. N. A complex trace fossil from the Spitskop Member (terminal Ediacaran–? Lower Cambrian) of southern Namibia. *Geological Magazine* **142,** 561–569 (2005).
19. Darroch, S. A. F. *et al.* Biotic replacement and mass extinction of the Ediacara biota. *Proc. R. Soc. B* **282,** 1814 (2015).
20. Edmonds, H. N. & German, C. R. Particle geochemistry in the Rainbow hydrothermal plume, Mid-Atlantic Ridge. *Geochimica et Cosmochimica Acta* **68,** 759–772 (2004).
21. de Baar, H. J. ., German, C. R., Elderfield, H. & van Gaans, P. Rare earth element distributions in anoxic waters of the Cariaco Trench. *Geochimica et Cosmochimica Acta* **52,** 1203–1219 (1988).
22. De Carlo, E. H. & Green, W. J. Rare earth elements in the water column of Lake Vanda, McMurdo Dry Valleys, Antarctica. *Geochimica et Cosmochimica Acta* **66,** 1323–1333 (2002).
23. Moffett, J. W. Microbially mediated cerium oxidation in sea water. *Nature* **345,** 421–423 (1990).
24. Sholkovitz, E. R., Landing, W. M. & Lewis, B. L. Ocean particle chemistry: The fractionation of rare earth elements between suspended particles and seawater. *Geochimica et Cosmochimica Acta* **58,** 1567–1579 (1994).
25. German, C. R. & Elderfield, H. Rare earth elements in the NW Indian Ocean. *Geochimica et Cosmochimica Acta* **54,** 1929–1940 (1990).

26. De Carlo, E. R. Rare earth element fractionation in hydrogenetic Fe-Mn crusts: The influence of carbonate complexation and phosphatization on Sm/Yb ratios. *Society for Sedimentary Geology* **66**, 271-285 (2000).
27. Planavsky, N. *et al.* Rare Earth Element and yttrium compositions of Archean and Paleoproterozoic Fe formations revisited: New perspectives on the significance and mechanisms of deposition. *Geochimica et Cosmochimica Acta* **74,** 6387–6405 (2010).
28. Bau, M. Scavenging of dissolved yttrium and rare earths by precipitating iron oxyhydroxide: experimental evidence for Ce oxidation, Y-Ho fractionation, and lanthanide tetrad effect. *Geochimica et Cosmochimica Acta* **63,** 67–77 (1999).
29. Ohta, A. & Kawabe, I. REE (III) adsorption onto Mn dioxide (MnO_2) and Fe oxyhydroxide: Ce (III) oxidation by MnO_2. *Geochimica et Cosmochimica Acta* **65,** 695–703 (2001).
30. Koeppenkastrop, D. & De Carlo, E. H. Sorption of rare-earth elements from seawater onto synthetic mineral particles: An experimental approach. *Chemical Geology* **95,** 251–263 (1992).
31. Quinn, K. A., Byrne, R. H. & Schijf, J. Sorption of yttrium and rare earth elements by amorphous ferric hydroxide: Influence of solution complexation with carbonate. *Geochimica et Cosmochimica Acta* **70,** 4151–4165 (2006).
32. Bau, M. & Koschinsky, A. Oxidative scavenging of cerium on hydrous Fe oxide: evidence from the distribution of rare earth elements and yttrium between Fe oxides and Mn oxides in hydrogenetic ferromanganese crusts. *Geochemical Journal* **43,** 37–47 (2009).

33. Bau, M. *et al.* Discriminating between different genetic types of marine ferro-manganese crusts and nodules based on rare earth elements and yttrium. *Chemical Geology* **381,** 1–9 (2014).
34. Haley, B. A., Klinkhammer, G. P. & McManus, J. Rare earth elements in pore waters of marine sediments. *Geochimica et Cosmochimica Acta* **68,** 1265–1279 (2004).
35. Ling, H.-F. *et al.* Cerium anomaly variations in Ediacaran–earliest Cambrian carbonates from the Yangtze Gorges area, South China: implications for oxygenation of coeval shallow seawater. *Precambrian Research* **225,** 110–127 (2013).
36. Nothdurft, L. D., Webb, G. E. & Kamber, B. S. Rare earth element geochemistry of Late Devonian reefal carbonates, Canning Basin, Western Australia: confirmation of a seawater REE proxy in ancient limestones. *Geochimica et Cosmochimica Acta* **68,** 263–283 (2004).
37. Webb, G. E. & Kamber, B. S. Rare earth elements in Holocene reefal microbialites: a new shallow seawater proxy. *Geochimica et Cosmochimica Acta* **64,** 1557–1565 (2000).
38. Slack, J., Grenne, T., Bekker, A., Rouxel, O. & Lindberg, P. Suboxic deep seawater in the late Paleoproterozoic: evidence from hematitic chert and iron formation related to seafloor-hydrothermal sulfide deposits, central Arizona, USA. *Earth and Planetary Science Letters* **255,** 243–256 (2007).
39. Goldstein, S. J. & Jacobsen, S. B. Rare earth elements in river waters. *Earth and Planetary Science Letters* **89,** 35–47 (1988).

40. Greaves, M. J., Statham, P. J. & Elderfield, H. Rare earth element mobilization from marine atmospheric dust into seawater. *Marine Chemistry* **46,** 255–260 (1994).

41. Johannesson, K. H., Lyons, W. B. & Bird, D. A. Rare earth element concentrations and speciation in alkaline lakes from the western U.S.A. *Geophys. Res. Lett.* **21,** 773–776 (1994).

42. Möller, P. & Bau, M. Rare-earth patterns with positive cerium anomaly in alkaline waters from Lake Van, Turkey. *Earth and Planetary Science Letters* **117,** 671–676 (1993).

43. Kaufman, A. J., Jacobsen, S. B. & Knoll, A. H. The Vendian record of Sr and C isotopic variations in seawater: Implications for tectonics and paleoclimate. *Earth and Planetary Science Letters* **120,** 409–430 (1993).

44. Bau, M., Tepe, N. & Mohwinkel, D. Siderophore-promoted transfer of rare earth elements and iron from volcanic ash into glacial meltwater, river and ocean water. *Earth and Planetary Science Letters* **364,** 30–36 (2013).

45. Kraemer, D., Kopf, S. & Bau, M. Oxidative mobilization of cerium and uranium and enhanced release of 'immobile' high field strength elements from igneous rocks in the presence of the biogenic siderophore desferrioxamine B. *Geochimica et Cosmochimica Acta* **165,** 263–279 (2015).

46. Kraemer, S. M. Iron oxide dissolution and solubility in the presence of siderophores. *Aquat. Sci.* **66,** 3–18 (2004).

47. Abbott, A. N., Haley, B. A., McManus, J. & Reimers, C. E. The sedimentary flux of dissolved rare earth elements to the ocean. *Geochimica et Cosmochimica Acta* **154,** 186–200 (2015).

48. Soyol-Erdene, T.-O. & Huh, Y. Rare earth element cycling in the pore waters of the Bering Sea Slope (IODP Exp. 323). *Chemical Geology* **358,** 75–89 (2013).
49. Freslon, N. *et al.* Rare earth elements and neodymium isotopes in sedimentary organic matter. *Geochimica et Cosmochimica Acta* **140,** 177–198 (2014).
50. Froelich, P. N. *et al.* Early oxidation of organic matter in pelagic sediments of the eastern equatorial Atlantic: suboxic diagenesis. *Geochimica et Cosmochimica Acta* **43,** 1075–1090 (1979).
51. Dromgoole, E. L. & Walter, L. M. Iron and manganese incorporation into calcite : effects of growth kinetics, temperature and solution chemistry. *Chemical geology* **81,** 311–336 (1990).
52. Bayon, G. *et al.* Evidence for intense REE scavenging at cold seeps from the Niger Delta margin. *Earth and Planetary Science Letters* **312,** 443–452 (2011).
53. De Baar, H. J. W., Bacon, M. P. & Brewer, P. G. Rare-earth distributions with a positive Ce anomaly in the Western North Atlantic Ocean. *Nature* **301,** 324–327 (1983).
